# Anti-Inflammatory Effects of Curvularin-Type Metabolites from a Marine-Derived Fungal Strain *Penicillium* sp. SF-5859 in Lipopolysaccharide-Induced RAW264.7 Macrophages

**DOI:** 10.3390/md15090282

**Published:** 2017-09-02

**Authors:** Tran Minh Ha, Wonmin Ko, Seung Jun Lee, Youn-Chul Kim, Jae-Young Son, Jae Hak Sohn, Joung Han Yim, Hyuncheol Oh

**Affiliations:** 1 College of Pharmacy, Wonkwang University, Iksan 54538, Korea; minhha19@outlook.com (T.M.H.); rabis815@naver.com (W.K.); daea5@naver.com (S.J.L.); yckim@wku.ac.kr (Y.-C.K.); 2 College of Medical and Life Sciences, Silla University, Busan 46958, Korea; bnm707@hanmail.net (J.-Y.S.); jhsohn@silla.ac.kr (J.H.S.); 3 Korea Polar Research Institute, KORDI, 7-50 Songdo-dong, Yeonsu-gu, Incheon 21990, Korea; jhyim@kopri.re.kr

**Keywords:** curvularin-type metabolites, marine-derived fungus, anti-inflammatory effects, nuclear factor kappa B

## Abstract

Chemical study on the extract of a marine-derived fungal strain *Penicillium* sp. SF-5859 yielded a new curvularin derivative (**1**), along with eight known curvularin-type polyketides (**2**–**9**). The structures of these metabolites (**1**–**9**) were established by comprehensive spectroscopic analyses, including 1D and 2D nuclear magnetic resonance (NMR) spectroscopy, and mass spectrometry (MS). In vitro anti-inflammatory effects of these metabolites were evaluated in lipopolysaccharide (LPS)-stimulated RAW264.7 macrophages. Among these metabolites, **3**–**9** were shown to strongly inhibit LPS-induced overproduction of nitric oxide (NO) and prostaglandin E_2_ (PGE_2_) with IC_50_ values ranging from 1.9 μM to 18.1 μM, and from 2.8 μM to 18.7 μM, respectively. In the further evaluation of signal pathways involved in these effects, the most active compound, (10*E*,15*S*)-10,11-dehydrocurvularin (**8**) attenuated the expression of inducible NO synthase (iNOS) and cyclooxygenase-2 (COX-2) in LPS-stimulated RAW264.7 macrophages. Furthermore, compound **8** was shown to suppress the upregulation of pro-inflammatory mediators and cytokines via the inhibition of the nuclear factor-κB (NF-κB) signaling pathway, but not through the mitogen-activated protein kinase (MAPK) pathway. Based on the comparisons of the different magnitude of the anti-inflammatory effects of these structurally-related metabolites, it was suggested that the opening of the 12-membered lactone ring in curvularin-type metabolites and blocking the phenol functionality led to the significant decrease in their anti-inflammatory activity.

## 1. Introduction

Inflammation is a vital part of the body’s immune response. There are two types of signals in the inflammatory process: the signals that initiate and maintain inflammation and the other signals that cease the process. Cellular and tissue damages are caused by the asymmetry of these two signals [1]. Macrophages are innate immune cells that play a critical role in the initiation, maintenance, and resolution of inflammation. These cells participate in tissue homeostasis, immune responses, and inflammations in lymphoid organs, liver, lung, and the central nervous system [2]. Stimulation of macrophages with stimuli such as lipopolysaccharide (LPS) induces a variety of pro-inflammatory cytokines and enzymes such as interleukins (ILs), tumor necrosis factor-α (TNF-α), inducible nitric oxide synthase (iNOS), and cyclooxygenase-2 (COX-2) [3]. Due to these significant physiological and pathophysiological roles, these cells are involved in a variety of inflammatory conditions such as rheumatoid arthritis, autoimmune and primary immunodeficiency diseases.

Secondary metabolites from marine-derived fungi have been proven to be an attractive resource for novel bioactive natural products as some of these metabolites possess biological activities of importance in medicine and agriculture [4,5]. Of note, marine microbes have been speculated to produce anti-inflammatory factors as their evolutionary strategy [6]. In the course of our continuing search for bioactive secondary metabolites from marine-derived fungi [7,8,9], we investigated the chemistry of the EtOAc extract obtained from the culture of a marine-derived fungal strain *Penicillium* sp. SF-5859, leading to the isolation of nine curvularin-type metabolites including a new analogue by the application of various chromatographic steps. This study describes the isolation and structural elucidation of these curvularin-type metabolites (**1**–**9**), including a new curvularin derivative (**1**), along with their anti-inflammatory effects in LPS-stimulated RAW264.7 macrophages.

## 2. Results and Discussion

**Figure 1:**
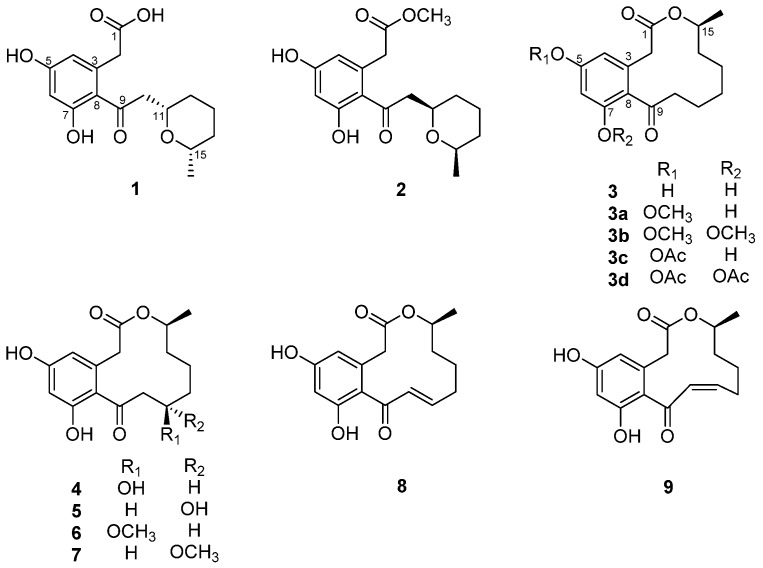
Structures of compounds **1**–**9**.

The structures of known polyketides (**2**–**9**) were determined by the analysis of their NMR, MS, and specific rotation data, along with comparisons of these data with those of the previously-published values in the literature. They were identified as curvulone B (**2**) [10], curvularin (**3**) [11], (11*R*,15*S*)-11-hydroxycurvularin (**4**) [12], (11*S*,15*S*)-11-hydroxycurvularin (**5**) [12], (11*R*,15*S*)-11-methoxycurvularin (**6**) [13], (11*S*,15*S*)-11-methoxycurvularin (**7**) [13], (10*E*,15*S*)-10,11-dehydrocurvularin (**8**) [12], and (10*Z*,15*S*)-10,11-dehydrocurvularin (**9**) [14] (Figure 1).

Among the curvularin-type fungal polyketides with a 12-membered lactone ring, the absolute configuration of the C-15 chiral center adjacent to the lactone oxygen is known to be variable depending on the reduction pathway by β-ketoacyl reductase in fungal macrolide biosynthesis. Until now, most of the C-15 chiral centers in this class have been described to have 15*S* configuration with the exception of curvalarins isolated from two marine fungal strains belonging to the genus *Curvularia* sp. [10,12]. On the basis of synthetic studies and analysis of chiroptical data of several curvularins, the absolute configuration 15*S* has been correlated with the negative specific rotations [12]. Therefore, the absolute configurations of the isolated metabolites (**2**–**9**) were considered to belong to the 15*S* series of curvularin-type metabolites based on the observation of negative signs of their specific rotation values (Supplementary Materials, Table S1). It is noteworthy that (10*Z*,15S*)-10,11-dehyrocurvularin that has the same planar structure as that of **9** was previously isolated from a hybrid strain derived from *Penicillium* sp. with a positive specific rotation {${\left[\alpha \right]}_{D}^{22}$ = +7.3 (c 0.78, EtOH)} [14], whereas **9** has a negative specific rotation ${\left[\alpha \right]}_{D}^{20}$ = −19.9 (*c* = 0.15, EtOH). Considering the relationship between the sign of the specific rotation and the absolute configuration at C-15, it was suggested that **9** is the first report of naturally-occurring (10*Z*,15*S*)-10,11-dehyrocurvularin, and the previously-reported (10*Z*,15S*)-10,11-dehyrocurvularin would have been a enantiomer of **9**.

Compound **1** was isolated as a colorless oil with an optical rotation of +13.6 (*c* = 0.22, EtOH). Its molecular formula was determined as C_16_H_20_O_6_ based on the observation of a sodium adduct ion at *m*/*z* 331.1158 [M + Na]^+^ and a protonated molecular ion at *m*/*z* 309.1336 [M + H]^+^ in its positive ion mode of HRESIMS. In the ^1^H-NMR spectrum of **1** recorded in CDCl_3_, the broad singlet signals for two aromatic protons and two oxymethine proton signals, and a signal for a methyl group that is coupled to the one of oxymethine protons were clearly observed. Analysis of the ^13^C-NMR and DEPT data of **1** suggested the presence of 16 carbons, including two sp^3^ oxymethines, two sp^2^ methine carbons, two carbonyl carbons, and four sp^2^ quaternary carbons. The signals for the four remaining methylene carbons and a methyl carbon were also observed in the ^13^C-NMR spectrum of **1**. Based on these structural features, including the molecular formula, scrutiny of the literature revealed that ^13^C-NMR data of **1** closely matched with the previously-published ^13^C-NMR chemical shift values for curvulone B [10]. Although ^1^H-NMR data of **1** recorded in CDCl_3_ contained some overlapped signals hampering direct comparison of the data with that of curvulone B, the ^1^H-NMR spectrum of **1** recorded in acetone-*d*_6_ (Table 1) pointed out that the chemical shifts of **1** were in close agreement with those of **2**, except for missing the methoxy signal in curvulone B.

Therefore, compound **1** was suggested to be a new analogue of curvulone B, having the carboxylic acid functionality instead of the methyl ester group in curvulone B. The relative configuration of the tetrahydropyran ring in **1** was deduced from the NOESY spectrum (Supplementary Materials, Figure S5). NOESY cross-peaks observed between H-11 and H-15 corroborated the *cis*-orientation of the substituents at C-11 and C-15. Thus, the relative configuration at C-11 and C-15 was proposed to be the same as that in curvulone B, whose absolute configuration was reported as 11*R* and 15*R* [10]. The specific rotation of **1** showed the opposite sign although the reported specific rotation (−15.24, *c* = 0.18, EtOH) of curvulone B [10] was in agreement with that of **1** (+13.6, *c* = 0.22, EtOH) regarding its magnitude. Therefore, the absolute configuration of **1** was assigned as 11*S* and 15*S*. As a result, **1** was determined as a new analogue of curvularin-type macrolides, and named curvulone C.

All isolated compounds (**1**–**9**) were evaluated for their anti-inflammatory effects. In the treatment of inflammatory disorders, the pharmacological manipulation of Nitric oxide (NO) and prostaglandin E_2_ (PGE_2_) overproduction has been suggested [15,16,17]. NO is a small molecule which is an intracellular mediator produced in various immune cells. It plays a pivotal role in the physiological and pathological condition of inflammatory symptoms [17]. PGE_2_ can also regulate the immune and inflammatory responses [15]. In this study, RAW264.7 macrophages were pre-treated for 3 h in the medium containing non-toxic concentrations (Table 2) of each compound (**1**–**9**), and then LPS (1 μg/mL) was treated for 24 h. According to the LPS stimulation of RAW264.7 macrophages, production of NO and PGE_2_ was increased, and the effects of all compounds on the production level of NO and PGE_2_ were evaluated by the Griess reaction and a PGE_2_ kit, respectively. As a result, compounds **3**–**9** dose-dependently inhibited the LPS-induced production of NO and PGE_2_, and their IC_50_ values are shown in Table 2. Based on the comparison of IC_50_ values for compounds **1**–**9**, it was evident that curvularin-type metabolites exhibit structure-dependent anti-inflammatory properties. Noticeably, the presence of a 12-membered macrolactone ring was essential for their anti-inflammatory activity as indicated by the observation that **1** and **2**, which are supposed to be derived from the cleavage of the lactone bond in the macrolactone ring, appeared to lose the inhibitory activity. To evaluate the impact of phenol and resorcinol groups in the curvularin-type metabolites for the anti-inflammatory activity, 5-*O*-methyl and 5,7,-di-*O*-methyl derivatives of curvularin (**3a** and **3b**, respectively), as well as 5-*O*-acetyl and 5,7,-di-*O*-acetyl derivatives of curvularin (**3c** and **3d**, respectively), were prepared, and the structures of **3a**–**3d** were identified by comparison the ^1^H and ^13^C-NMR spectroscopic data with those of curvularin (**3**), and confirmed by their HRESIMS. These compounds exhibited the similar chemical shifts, except for the additional appearances of the methoxy signals and acetyl signals. The position of the methoxy group in **3a** was confirmed by HMBC correlation between methoxy signal at δ_H_ 3.77 (3H, s, 5-OCH_3_) and δ_C_ 161.8 (C-5). The NOESY cross-peaks between acetyl proton (5-OCH_3_) with H-2, H-4, and H-16 of **3c** were used to identify the position of the acetyl group in **3c**. According to the anti-inflammatory effects of **3a**–**3d**, as shown in Table 2, the blocking of the phenol and resorcinol groups appeared to lead to the significant decrease in the anti-inflammatory activity, implying the crucial role of the intact phenols of curvularin-type metabolites for their anti-inflammatory activity. However, this observation is inconsistent with the previous study reporting that **3d** showed higher inhibitory effect on the cytokine-mediated induction of the iNOS promotor than that of (*S*)-curvularin (**3**) in the assay of human iNOS promotor activity in human alveolar epithelial A549/8 cells [11]. Different cell types employed in both studies may have resulted in these inconsistent biological effects. In addition, the present study showed that the modification of the aliphatic part around the macrolactone ring could influence the anti-inflammatory activity of curvularin-type metabolites as shown that unsaturated (*S*)-curvularin-derivatives (**8** and **9**) and 11-methoxy and 11-hydroxy derivatives (**4**–**7**) possess stronger anti-inflammatory activities than that of **3**. Further systematic investigations are needed to clarify the structure activity relationship associated with the modification of the aliphatic part of the molecule.

**Figure 2:**
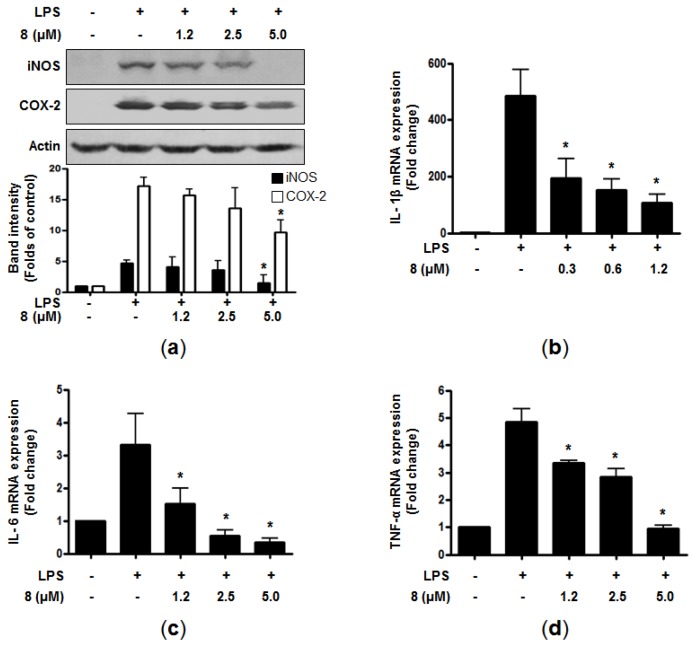
Effects of **8** on protein expression levels of iNOS and COX-2 (**a**), and mRNA expression levels of IL-1β (*Il1b*) (**b**), IL-6 (*Il6*) (**c**), and TNF-α (*Tnf*) (**d**) in RAW264.7 macrophages. The cells were pre-treated for 3 h with the indicated concentrations of **8** and stimulated for 24 h (**a**), and 6 h (**b**–**d**) with LPS (1 μg/mL). The measurement of western blot analysis and RNA quantification for *Il1b*, *Il6*, and *Tnf* expression were performed as described in the Materials and Methods. Representative data from three independent experiments were shown. * *p* < 0.05 compared with the group treated with LPS.

Among the curvularin-type metabolites encountered in this study, compound **8** was identified as the most active anti-inflammatory metabolite based on its IC_50_ values (Table 2). Therefore, we further tested whether inhibitory effects of **8** against NO and PGE_2_ productions are correlated with the protein expression of pro-inflammatory enzymes (i.e., iNOS and COX-2, respectively), which are known to catalyze the production of NO and PGEs in LPS-stimulated cells. When RAW264.7 macrophages were pretreated with the indicated concentrations of **8** for 3 h before stimulation with LPS for 24 h, the presence of **8** led to the attenuation of the excessive protein expression of iNOS and COX-2 in a dose-dependent manner (Figure 2a). Upon the stimulation by LPS, macrophages can also trigger the production of pro-inflammatory cytokines such as TNF-α, and ILs [18]. The overproduction of these cytokines contributes to the pathogenesis of inflammatory diseases [19,20,21]. Thus, we further evaluated the effects of **8** on the mRNA expression of pro-inflammatory cytokines in the LPS-induced cells. The cells were pre-treated with indicated concentration for 3 h, followed by LPS stimulation (1 μg/mL) for 6 h. The mRNA expression of pro-inflammatory cytokines was determined by RT-qPCR. As demonstrated in Figure 2b–d, compound **8** markedly suppressed the mRNA expression of IL-1β, IL-6, and TNF-α in a dose-dependent manner. These results indicated that **8** attenuated the gene expression of pro-inflammatory cytokines at the transcriptional level.

**Figure 3:**
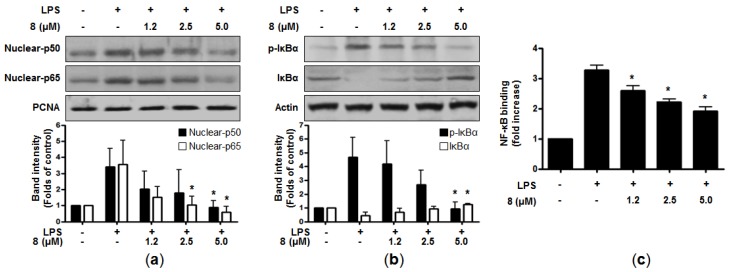
Effects of **8** on NF-κB activation (nuclear-p50 and p65) (**a**), IκBα phosphorylation and degradation (**b**), and the DNA binding activity of NF-κB (**c**) in LPS-treated RAW264.7 macrophages. Cells were pre-treated with the indicated concentrations of **8** for 3 h and stimulated with LPS (1 μg/mL) for 1 h. Western blot analysis (IκBα and p-IκBα in the cytoplasm and NF-κB in the nucleus) was performed as described in the Materials and Methods. Representative blots from three independent experiments are shown. A commercially-available NF-κB ELISA kit (Active Motif) was used to test the nuclear extracts and determine the degree of NF-κB binding. The data shown represent the mean values of three independent experiments. * *p* < 0.05 compared with the group treated with LPS.

It has been reported that many cellular signaling pathways and transcription factors are related to the expression of pro-inflammatory genes and enzymes in immune cells [18]. Nuclear factor-κB (NF-κB) is one of the important transcriptional factors involved in inflammation-related disorders. It is known to modulate inflammatory genes and the expression of pro-inflammatory mediators, such as iNOS and COX-2 [22]. In normal cells, NF-κB consists of inactive subunits of p50 and p65 bound to the inhibitor of NF-κB (IκB-α) [23]. The NF-κB signaling pathway can be activated by LPS or other stimuli, which then phosphorylates IκB-α, leading to degradation and subsequent translocation of NF-κB into the nucleus. The data from our study indicated that pre-treatment of **8** clearly suppressed the nuclear translocation of p50 and p65 in a dose-dependent manner (Figure 3a). In addition, when the cells were treated with LPS alone, the phosphorylation level of IκB-α was increased, whereas **8** attenuated this phosphorylation of IκB-α. Compound **8** also blocked the degradation of IκB-α in a concentration-dependent manner (Figure 3b). In line with these, LPS-induced DNA binding activity of NF-κB was declined in the nuclear extracts of the cells co-treated with **8** (Figure 3c). Taken together, it was suggested that **8** could suppress the induction of pro-inflammatory mediators and cytokines through the downregulation of the NF-κB signaling pathway.

**Figure 4:**
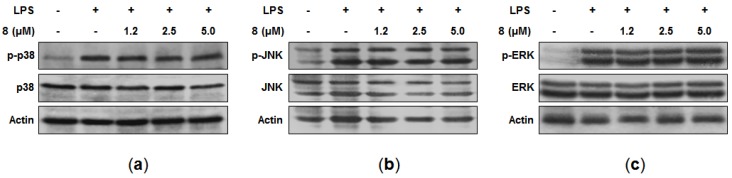
Effects of **8** on p38 (**a**), JNK (**b**), and ERK (**c**) phosphorylation in RAW264.7 macrophages. Cells were pre-treated with the indicated concentrations of **8** for 3 h and stimulated for 30 min with LPS (1 μg/mL). Cell extracts were subjected to Western blotting with antibodies specific for phosphorylated-p38 (p-p38), phosphorylated JNK (p-JNK), or phosphorylated ERK1/2 (p-ERK). Membranes were stripped and re-probed to measure the total abundance of each MAPK as a control measurement. Representative blots from three independent experiments are shown.

Mitogen-activated protein kinase (MAPK) pathways are also known to be involved in the expression of pro-inflammatory cytokines in macrophages [24]. Thus, the effects of **8** on the LPS-induced phosphorylation of MAPK were examined. Although the treatment of LPS for 30 min with the cells caused the phosphorylation of p38, c-Jun N-terminal kinase (JNK), and extracellular signal-regulated kinase (ERK), our data indicated that **8** did not suppress these phosphorylations (Figure 4a–c). Consequently, the anti-inflammatory effects of **8** did not seem to be mediated through MAPK signaling pathways, and further study is needed in order to elucidate the specific target of **8** involved in its anti-inflammatory activity.

Curvularin-type metabolites are macrocyclic lactones produced by several fungi of the genera *Curvularia*, *Penicillium*, and *Alternaria* [11,25]. They have been reported to possess a variety of biological activities. For example, curvularin (**3**) inhibited inducible transcription and synthesis of iNOS and COX-2 through blocking the activation of the transcription factor signal transducer and activator of transcription (STAT)-1α in human alveolar epithelial A549/8 cells [11,26]. The suppressive effect of **3** against the expression of various pro-inflammatory cytokines and chemokines was also observed in the mice model of chronic induced arthritis [27]. Recently, cytotoxicity of compounds **6**–**8** against a small panel of human tumor cell lines has been observed, and this was partially linked to their suppressive effect on the activation of the TNFα-induced NF-κB pathway [28]. It was also reported that **8** acts as a covalent inhibitor of p97, interfering with its ATPase activity [29]. Furthermore, the antibacterial activity inhibiting the growth of *E. coli* of 11-hydroxylcurvularin (**4**–**5**) was also demonstrated [30]. In the present study, nine curvularin-type metabolites, including a new curvularin derivative (**1**), were isolated from a marine-derived fungal strain *Penicillium* sp. SF-5859. From the evaluation of anti-inflammatory effects of these metabolites in LPS-stimulated RAW 264.7 cells that has not been reported previously, it was suggested that structural variation of curvularin-type metabolites could lead to the development of a promising agent for the treatment of inflammation-related diseases. In addition, this study demonstrated that the most active metabolite, (10*E*,15*S*)-10,11-dehydrocurvularin (**8**) can significantly suppress the induction of pro-inflammatory mediators and cytokines via inhibition of the NF-κB signaling pathway, and could play a role as a lead compound in the search for anti-inflammatory drugs.

## 3. Materials and Methods

### 3.1. General Experimental Procedures

Optical rotations were recorded using a Jasco P-2000 digital polarimeter (Jasco, Easton, PA, USA). NMR spectra (1D and 2D) were recorded in a JEOL JNM ECP-400 spectrometer (400 MHz for ^1^H and 100 MHz for ^13^C, JEOL Ltd., Akishima, Japan), and chemical shifts were referenced relative to the corresponding residual solvents signals (δ_H_ 2.05/δ_C_ 29.8 for acetone-*d*_6_, δ_H_ 7.26/δ_C_ 77.2 for CDCl_3_, and δ_H_ 3.30/δ_C_ 49.0 for CD_3_OD). HMQC and HMBC experiments were optimized for ^1^*J*_CH_ = 140 Hz and ^n^*J*_CH_ = 8 Hz, respectively. HRESIMS data were obtained using an ESI Q-TOF MS/MS system (AB SCIEX Triple, SCIEX, Framingham, MA, USA). Flash column chromatography was performed on silica gel (Kieselgel 60, 70–230 mesh and 230–400 mesh, Merck, Kenilworth, NJ, USA) and YMC octadecyl-functionalized silica gel (C_18_, YMC CO., Kyoto, Japan). YMC semiprep-C_18_ column (20 × 150 mm; 4 μm particle size; 80 Å pore size, 5 mL/min, YMC CO., Kyoto, Japan) and Shodex Ohpak SB 802.5 (8 × 300 mm; 6 μm particle size; 80 Å pore size, 0.6 mL/min, Showa Denko K.K., Tokyo, Japan) were used for HPLC (YoungLin, Anyang, Korea) separations. TLC was performed on Kieselgel 60 F_254_ (Merck, Kenilworth, NJ, USA) or reversed-phase (RP)-18 F_254s_ (Merck, Kenilworth, NJ, USA) plates. Spots were visualized by spraying with 10% aqueous H_2_SO_4_ solution, followed by heating. All compounds were detected by UV absorption at 210 and 254 nm.

RPMI1640, fetal bovine serum (FBS), and other tissue culture reagents were purchased from Gibco BRL Co. (Grand Island, NY, USA). All other chemicals were obtained from Sigma-Aldrich Co. (St. Louis, MO, USA). Primary antibodies (COX-2: sc-1745; iNOS: sc-650; IκB-α: sc-371; p-IκB-α: sc-8404; p50: sc-7178; p65: sc-8008, Santa Cruz Biotechnology, Dallas, TX, USA, p-ERK: #9101; ERK: #9102; p-JNK: #9251; JNK: #9252S; p-p38: #9211; p38: 9212S, Cell Signaling Technology, Danvers, MA, USA) and secondary antibodies (mouse: ap124p; goat: ap106p; rabbit: ap132p, Millipore, Billerica MA, USA). Enzyme-linked immunosorbent assay (ELISA) kits for PGE_2_ were purchased from R and D Systems, Inc. (Minneapolis, MN, USA).

### 3.2. Fungal Material and Fermentation

*Penicillium* sp. SF-5859 was isolated from an unidentified sponge that was collected using a dredge in the Ross Sea (76 06.25635 S 169 12.6752 E) on 8 February 2011. The surface of the sponge was sterilized, and one gram of the sample was ground with a mortar and pestle, followed by mixing with sterile seawater (10 mL). A portion (0.1 mL) of the sample was processed utilizing the spread plate method in potato dextrose agar (PDA) medium containing sterile seawater collected in the Busan area. The plate was incubated at 25 °C for 14 days. After subculturing the isolates several times, the final pure cultures were selected and preserved at −70 °C. The fungal strain SF-5859 was identified based upon the analysis of their ribosomal RNA (rRNA) sequences. A GenBank search with the 28S rRNA gene of SF-5859 (GenBank accession number KF745792) indicated *Penicillium chrysogenum* (FJ890400), *P. steckii* (HM469415), *P. paxilli* (FJ890408), and *P. citrinum* (JN938950), as the closest match showing sequence identities of 99.48%, 98.69%, 98.69%, and 98.43%, respectively. Therefore, the marine-derived fungal strain SF-5859 was characterized as *Penicillium* sp., but could not be definitively identified to a specific species.

### 3.3. Extraction and Isolation

The fungal strain *Penicillium* sp. SF-5859 was cultured on ten Fernbach-style flasks each containing 100 g of semi-solid vermiculite and 400 mL of PDB with 3% (*w*/*v*) NaCl. The flasks were individually inoculated with 2 mL seed cultures of the fungal strain and incubated at 25 °C for 14 days then extracted with EtOAc (4 L per one flask). The combined extract solutions were filtered through filter paper and evaporated to dryness resulting in a crude extract SF5859 (2.2 g). The crude extract was fractionated on reversed phase (RP) C_18_ flash column chromatography (5 × 30 cm), eluting with a stepwise gradient of 20, 40, 60, 80, and 100% (*v*/*v*) MeOH in H_2_O (500 mL each) to give six fractions, SF5859-1 to SF5859-6, consecutively. The fraction SF5859-3 was applied to a chromatographic column packed with silica gel (2 × 30 cm). The column was subsequently eluted with gradients of CH_2_Cl_2_ in EtOAc (8/1 *v*/*v*, 200 mL) and (4/1 *v*/*v*, 150 mL), to yield **8** (30.0 mg) and seven other fractions, SF5859-31 to SF5859-38, which were pooled based on TLC analysis. The fourth fraction, SF5859-34, was further purified by semi-preparative reverse-phase HPLC eluting with a gradient of MeOH (60% to 80% in 20 min) in water (0.1% HCOOH) to afford **9** (1.5 mg, *t*_R_ = 13.5 min) and **2** (0.5 mg, *t*_R_ = 19 min). Similarly, the sixth fraction SF5859-36 was subjected to semi-preparative RP HPLC column (50–80% MeOH in H_2_O (0.1% HCOOH) over 30 min), giving two sub-fractions, SF5859-361 and SF5859-362, and **6** (3.5 mg, *t*_R_ = 46 min) was isolated from the sub-fraction SF5859-362 by performing on Shodex Ohpak SB 802.5 HPLC column (30–75% MeOH in H_2_O over 50 min). The seventh fraction SF5859-37 was separated into **5** (1.5 mg, *t*_R_ = 28 min) and two other sub-fractions by using semi-preparative RP HPLC column (30–60% MeOH in H_2_O (0.1% HCOOH) in 30 min). Among these sub-fractions, SF5859-373 was further separated on semi-preparative RP HPLC column (40–65% MeOH in H_2_O (0.1% HCOOH) in 25 min), giving compound **4** (2.5 mg, *t*_R_ = 20.5 min). The eighth fraction SF5859-38 was separated firstly by C_18_ chromatographic column (1.5 × 20 cm), eluting with MeOH in H_2_O (1/3 *v*/*v*), and the sub-fraction SF5859-383 was further purified by semi-preparative RP HPLC column (30–55% CH_3_CN in H_2_O (0.1% HCOOH) over 25 min), yielding **1** (1.3 mg, *t*_R_ = 22 min). The fraction SF5859-4 was chromatographed on silica gel column (3 × 30 cm), eluting with CH_2_Cl_2_ in EtOAc (7/1 *v*/*v*). From this, the major metabolite **3** (450.0 mg) was obtained, along with four another fractions. The fifth fraction SF5859-45 was subjected to a final purification on semi-preparative RP HPLC column (60–75% MeOH in H_2_O (0.1% HCOOH) over 15 min) to afford **7** (2 mg, *t*_R_ = 13 min).

Curvulone C (**1**): colorless oil; ${\left[\alpha \right]}_{D}^{22}$ = +13.6 (c 0.22, EtOH); ^1^H-NMR data (CDCl_3_, acetone-*d*_6_, 400 MHz) and ^13^C-NMR data (CDCl_3_, 100 MHz), see Table 1; HRESIMS *m*/*z* 309.1336 [M + H]^+^ (calcd. for C_16_H_21_O_6_, 309.1338), 331.1158 [M + Na]^+^ (calcd. for C_16_H_20_O_6_Na, 331.1158).

### 3.4. Preparation of Compounds ***3a*** and ***3b***

*N*,*N*-diisopropylethylamine (50 μL) was added to a solution of curvularin (**3**, 15 mg) in 1 mL of MeOH, followed by the addition of TMSCHN_2_ (110 μL, 2.2 M in *n*-hexane). The reaction mixture was stirred for 15 h at room temperature. The solution was then concentrated in vacuo and extracted with EtOAc and H_2_O prior to the evaporation of organic phase. Subsequently, the residual material was subjected to semi-preparative RP HPLC eluting with the gradient of methanol in water (0.1% HCOOH) from 70% to 86% over 18 min to afford methylated products **3a** (4 mg, *t*_R_ = 14 min) and **3b** (6 mg, *t*_R_ = 16 min).

5-*O*-methylcurvularin (**3a**): white amorphous powder; ^1^H-NMR (CD_3_OD_,_ 400 MHz) and ^13^C-NMR data (CD_3_OD, 100 MHz), Supplementary Materials Figures S7 and S8; HRESIMS *m*/*z* 309.1667 [M + H]^+^ (calcd. for C_17_H_21_D_2_O_5_ due to deuterium exchange, 309.1671).

5,7-Di-*O*-methylcurvularin (**3b**): white amorphous powder; ^1^H-NMR (CD_3_OD, 400 MHz) and ^13^C-NMR data (CD_3_OD, 100 MHz), Supplementary Materials, Figures S13 and S14; HRESIMS *m*/*z* 321.1706 [M + H]^+^ (calcd. for C_18_H_25_O_5_, 321.1702).

### 3.5. Preparation of Compounds ***3c*** and ***3d***

Curvularin (**3**, 10 mg) was dissolved in 600 μL acetone, followed by the addition of acetic anhydride (600 μL). The reaction was started with adding a catalytic amount of *N*,*N*-dimethylpyridin-4-amine. The reaction mixture was stirred for 3 h at room temperature. The resulting solution was dried in vacuo then partitioned with EtOAc and H_2_O prior to the evaporation of the organic phase. Thereafter, the residual material was subjected to semi-preparative RP HPLC eluting with the gradient of methanol in water (0.1% HCOOH) from 62% to 80% over 19 min to afford the acetylated products **3c** (2 mg, *t*_R_ = 14 min) and **3d** (3.5 mg, *t*_R_ = 16 min).

5-*O*-acetylcurvularin (**3c**): white amorphous powder; ^1^H-NMR (acetone-*d*_6_, 400 MHz), Supplementary Materials, Figure S16; HRESIMS *m*/*z* 357.1336 [M + Na]^+^ (calcd. for C_18_H_22_NaO_6_, 357.1314).

5,7-Di-*O*-acetylcurvularin (**3d**): white amorphous powder; ^1^H-NMR (acetone-*d_6_*, 400 MHz), Supplementary Materials, Figure S19; HRESIMS *m*/*z* 399.1441 [M + Na]^+^ (calcd. for C_20_H_24_NaO_7_, 399.1420).

### 3.6. Cell Culture and Cytotoxic Assay

RAW264.7 macrophages were maintained at a density of 5 × 10^5^ cells/mL in RPMI1640 medium supplemented with 10% heat-inactivated FBS, penicillin G (100 units/mL), streptomycin (100 mg/mL), and L-glutamine (2 mM), and were incubated at 37 °C in a humidified atmosphere containing 5% CO_2_. For determination of cell viability, cells (1 × 10^5^ cells/well in 96-well plates) were incubated with 3-(4,5-dimethylthiazol-2-yl)-2,5-diphenyltetrazolium bromide (MTT) at a final concentration of 0.5 mg/mL for 3 h, and the formazan formed was dissolved in acidic 2-propanol. The optical density was measured at 540 nm with a microplate reader (BioRad, Hercules, CA, USA). The optical density of the formazan formed in control (untreated) cells was considered to represent 100% viability.

### 3.7. Determination of Nitrite and PGE_2_

As an indicator of NO production in RAW264.7 macrophages, production of nitrite, a stable end-product of NO oxidation, was estimated. Briefly, the concentration of nitrite in the conditioned media was determined by a method based on the Griess reaction [31], and the details of the assay were described previously [32].

### 3.8. Quantitative Real-Time Polymerase Chain Reaction (qRT-PCR)

Total RNA was isolated from RAW264.7 macrophages using Trizol (Invitrogen, Carlsbad, CA, USA) according to the manufacturer’s protocol and quantified spectrophotometrically at 260 nm. Total RNA (1 μg) was reverse-transcribed using the High Capacity RNA-to-cDNA kit from Applied Biosystems (Carlsbad, CA, USA). The cDNA was amplified using the SYBR Premix Ex Taq kit from TaKaRa Bio Inc. (Shiga, Japan) and a StepOnePlus Real-Time PCR system from Applied Biosystems. qRT-PCR was performed in a 20 μL total volume of 0.8 μM of each primer, 2.5 μL of cDNA sample, diethyl pyrocarbonate (DEPC)-treated water, and 10 μL SYBR Green PCR Master Mix. The primer sequences were designed using Primer Quest software from Integrated DNA Technologies (Cambridge, MA, USA). The primer sequences used in this study were provided previously [32]. The optimal conditions for PCR amplification of cDNA were established using the manufacturer’s instructions. In addition, the data were analyzed using StepOne software from Applied Biosystems (Carlsbad, CA, USA). The cycle number at the linear amplification threshold (Ct) values for the endogenous control Glyceraldehyde 3-phosphate dehydrogenase (GAPDH) and the target gene were recorded. Relative gene expression (target gene expression normalized to the expression of the endogenous control gene) was calculated using the comparative Ct method (2^−ΔΔCt^).

### 3.9. Western Blot Analysis

The proteins of iNOS, COX-2, NF-κB, and MAPKs were measured by Western blot analysis. The details of procedures for Western blot analysis were described previously [32].

### 3.10. Preparation of Nuclear and Cytosolic Fractions

The proteins of nuclear NF-κB (p50 and p65) and cytosolic IκBα (p-IκBα and IκBα) were obtained by using cytosolic and nuclear fractions. The details of this procedure have been described previously [32].

### 3.11. DNA Binding Activity of NF-κB

TransAM kit (Active Motif, Carlsbad, CA, USA) was used to estimate the DNA-binding activity of NF-κB in the nuclear extract according to the manufacturer’s instructions as described previously [32].

### 3.12. Statistical Analysis

Data were presented as the mean ± standard deviation (S.D.) of at least three independent experiments. One-way analysis of variance (ANOVA), followed by Tukey’s multiple comparison tests, was used to compare three or more groups. Statistical analysis was performed using GraphPad Prism software, version 4.00 (GraphPad Software Inc., San Diego, CA, USA).

## Figures and Tables

**Table 1:** ^1^H and ^13^C-NMR spectroscopic data for curvulone C (**1**).

Position	δ_C_^a,c^, Type	δ_H_^a,d^ (*J* in Hz)	δ_H_^b,d^ (*J* in Hz)
1	172.6, C	-	-
2	42.1, CH_2_	3.51–3.59 ^e^	3.55, d (15.6)
		3.51–3.59 ^e^	3.66, d (15.6)
3	136.0, C	-	-
4	111.8, CH	6.35 (s)	6.32, s
5	161.5, C	-	-
6	105.0, CH	6.47 (s)	6.35, s
7	160.5, C	-	-
8	120.5, C	-	-
9	207.3, C	-	-
10	48.6, CH_2_	3.39, m	3.08, dd (15.2, 8.4)
		2.61, br	2.93, dd (15.2, 4.8)
11	78.7, CH	4.16, br	3.89, m
12	30.7, CH_2_	1.44–1.71 ^e^	1.54 ^e^
		1.44–1.71 ^e^	1.65, brd (13.2)
13	32.7, CH_2_	1.44–1.71 ^e^	1.22, m
		1.88, m	1.77, m
14	23.1, CH_2_	1.27, m	1.10, m
		1.44–1.71 ^e^	1.54 ^e^
15	75.4, CH	3.51–3.59 ^e^	3.38, m
16	21.5, CH_3_	1.18, d (6.0)	1.04, d (6.0)
-OH	-	9.70, br	-

^a^ Recorded in CDCl_3_. ^b^ Recorded in acetone-*d*_6_. ^c^ 100 MHz. ^d^ 400 MHz. ^e^ Overlapped signals.

**Table 2:** Inhibitory Effects of **1**–**9** and **3a**–**3d** against NO and PGE_2_ production in LPS-treated RAW 264.7 macrophages.

Compounds	IC_50_ (μM)		Cytotoxicity (μM) ^a^
	NO	PGE_2_	
**1**	>80	>80	>80
**2**	>80	>80	>80
**3**	18.1 ± 5.2	18.7 ± 4.9	40
**3a**	>80	40.2 ± 5.1	>80
**3b**	60.6 ± 16.4	49.4 ± 14.0	>80
**3c**	46.9 ± 3.7	73.7 ± 17.1	>80
**3d**	77.5 ± 9.3	>80	>80
**4**	11.5 ± 2.7	15.6 ± 5.2	40
**5**	7.2 ± 1.6	14.1 ± 4.0	40
**6**	2.6 ± 0.4	3.0 ± 1.3	20
**7**	3.5 ± 0.5	6.0 ± 1.9	20
**8**	1.9 ± 0.3	2.7 ± 0.4	20
**9**	4.4 ± 0.8	6.2 ± 1.1	20

^a^ The maximum concentration not affecting cell viability.

## Supplementary Materials

The following are available online at www.mdpi.com/1660-3397/15/9/282/S1, **Figures S1–S19**. NMR spectra of **1** and **3a**–**3d**; **Table S1**. The optical rotation values of **1**–**9** in comparison with the published values.
