# Supplementary material for: Anti-Inflammatory Effects of Curvularin-Type Metabolites from a Marine-Derived Fungal Strain Penicillium sp. SF-5859 in Lipopolysaccharide-Induced RAW264.7 Macrophages
Source: Mar Drugs. 2017 Sep 2;15(9):282. doi: 10.3390/md15090282 (PMC5618421; doi:10.3390/md15090282)
Supplement: Supplementary file 1 [file marinedrugs-15-00282-s001.pdf]

Supplementary Materials

Anti-Inflammatory Effects of Curvularin-Type Metabolites from a Marine-Derived Fungal Strain *Penicillium* sp. SF-5859 in Lipopolysaccharide-Induced RAW264.7 Macrophages

Tran Minh Ha [1], Wonmin Ko [1], Seung Jun Lee [1], Youn-Chul Kim [1], Jae-Young Son [2], Jae Hak Sohn [2], Joung Han Yim [3] and Hyuncheol Oh [1,*]

[1] College of Pharmacy, Wonkwang University, Iksan 54538, Korea; minhha19@outlook.com (T.M.H.); rabis815@naver.com (W.K.); daea5@naver.com (S.J.L.); yckim@wku.ac.kr (Y.-C.K.)

[2] College of Medical and Life Sciences, Silla University, Busan 46958, Korea; bnm707@hanmail.net (J.-Y.S.); jhsohn@silla.ac.kr (J.H.S.)

[3] Korea Polar Research Institute, KORDI, 7-50 Songdo-dong, Yeonsu-gu, Incheon 21990, Korea; jhyim@kopri.re.kr

* Correspondence: hoh@wku.ac.kr; Tel.: +82-63-850-6815

List of supporting information

Figure/Table	Content	Page
**Figure S1**	HRESI mass spectrum of compound **1**	S1
**Figure S2**	^{1}H NMR spectrum (400 MHz, acetone-*d6*) of compound **1**	S2
**Figure S3**	^{1}H NMR spectrum (400 MHz, $CDCl_3$) of compound **1**	S3
**Figure S4**	^{13}C NMR spectrum (100 MHz, $CDCl_3$) of compound **1**	S4
**Figure S5**	NOESY spectrum of compound **1**	S5
**Figure S6**	HRESI mass spectrum of compound **3a**	S6
**Figure S7**	^{1}H NMR spectrum (400 MHz, CD_3OD) of compound **3a**	S7
**Figure S8**	^{13}C NMR spectrum (100 MHz, CD_3OD) of compound **3a**	S8
**Figure S9**	HMQC spectrum of compound **3a**	S9
**Figure S10**	COSY spectrum of compound **3a**	S10
**Figure S11**	HMBC spectrum of compound **3a**	S11
**Figure S12**	HRESI mass spectrum of compound **3b**	S12
**Figure S13**	^{1}H NMR spectrum (400 MHz, CD_3OD) of compound **3b**	S13
**Figure S14**	^{13}C NMR spectrum (100 MHz, CD_3OD) of compound **3b**	S14

**Figure S15**	HRESI mass spectrum of compound **3c**	S15
**Figure S16**	^{1}H NMR spectrum (400 MHz, acetone-*d6*) of compound **3c**	S16
**Figure S17**	NOESY spectrum of compound **3c**	S17
**Figure S18**	HRESI mass spectrum of compound **3d**	S18
**Figure S19**	^{1}H NMR spectrum (400 MHz, $CDCl_3$) of compound **3d**	S19
**Table S1**	The optical rotation values of **1-9** in comparison with the published values.	S20

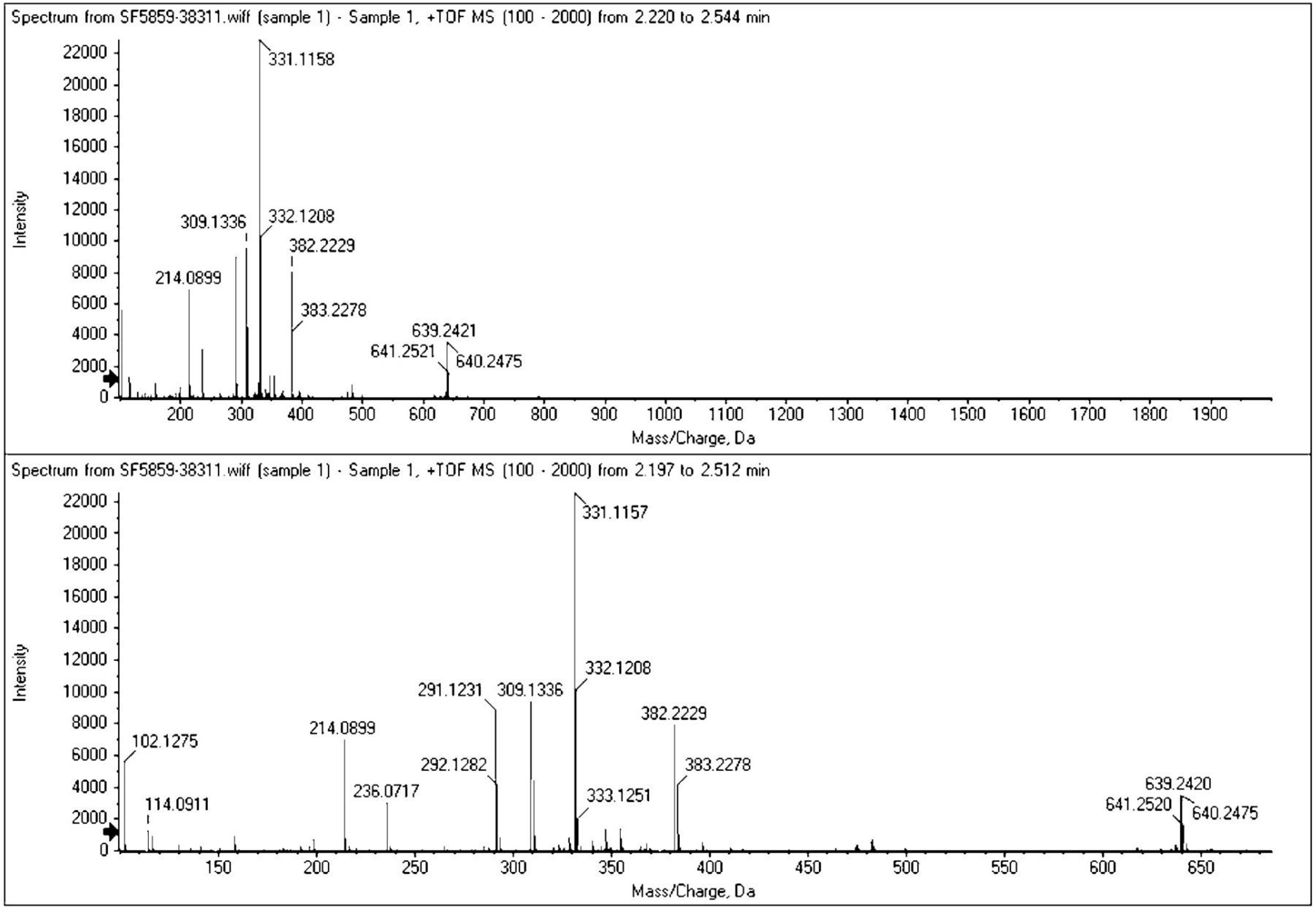

Figure S1. HRESI mass spectrum of compound **1**

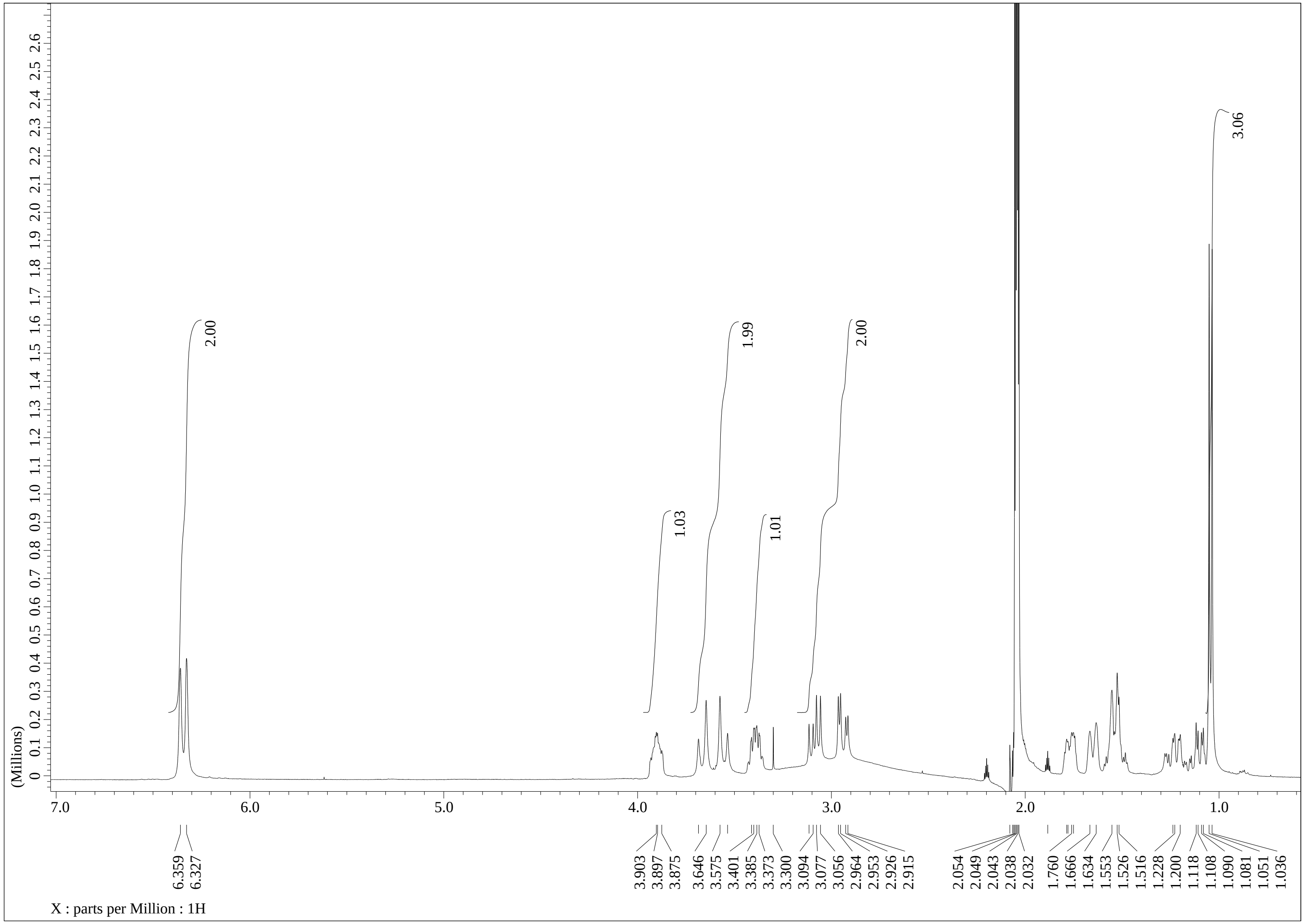

Figure S2. ^{1}H NMR spectrum of **1** recorded in acetone-*d6*

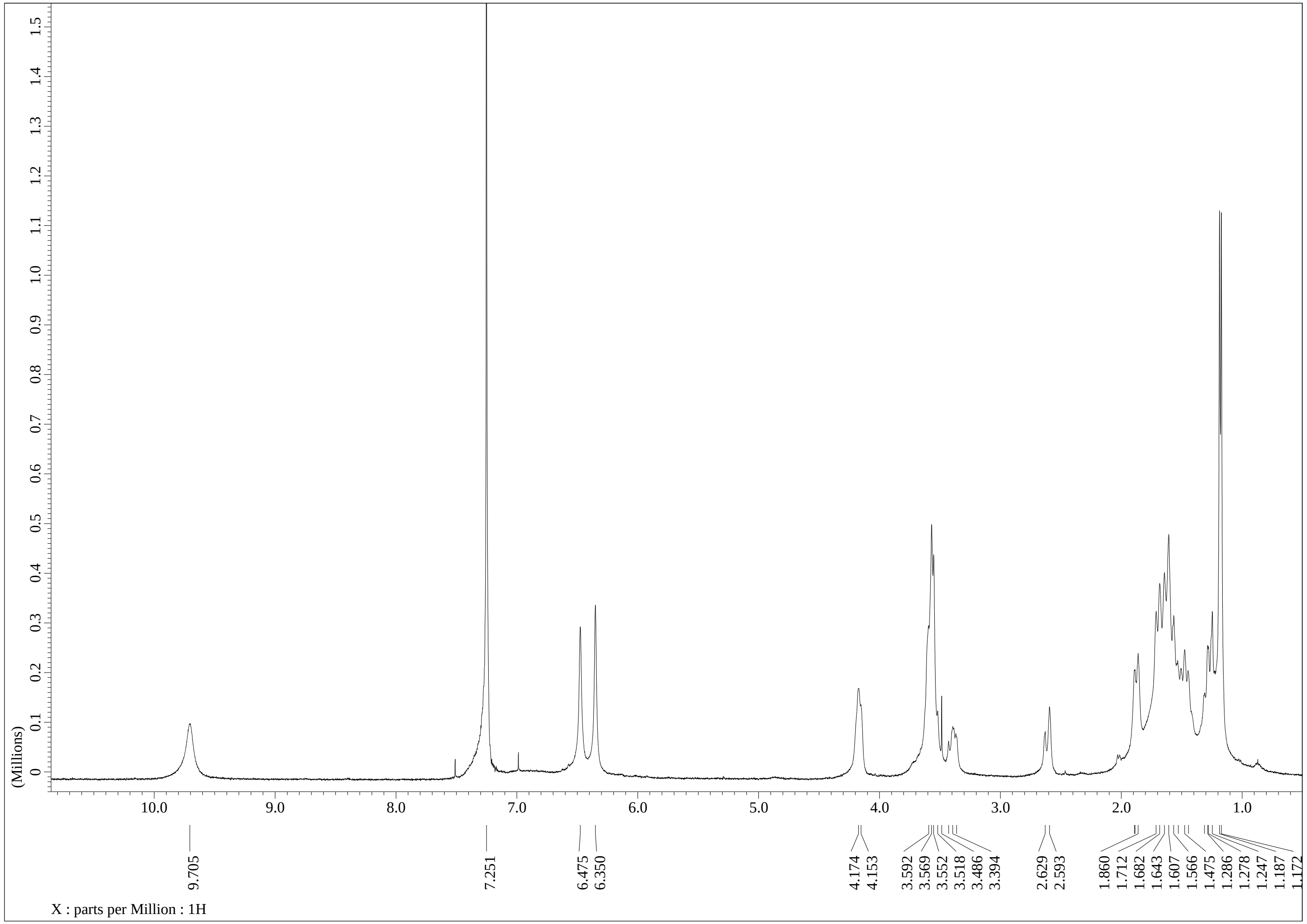

Figure S3. ^{1}H NMR spectrum of **1** recorded in $CDCl_3$

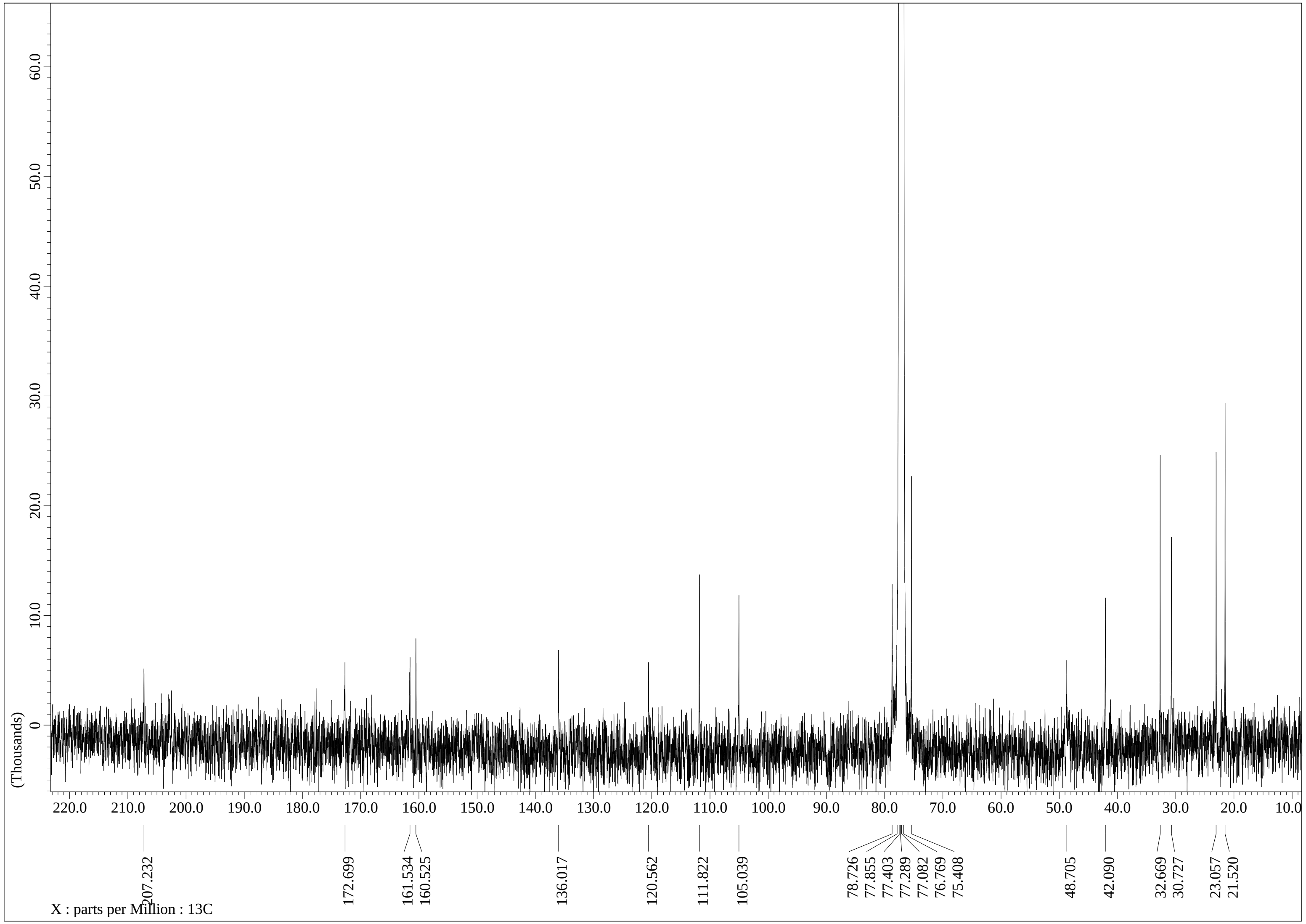

Figure S4. ^{13}C NMR spectrum of **1** recorded in $CDCl_3$

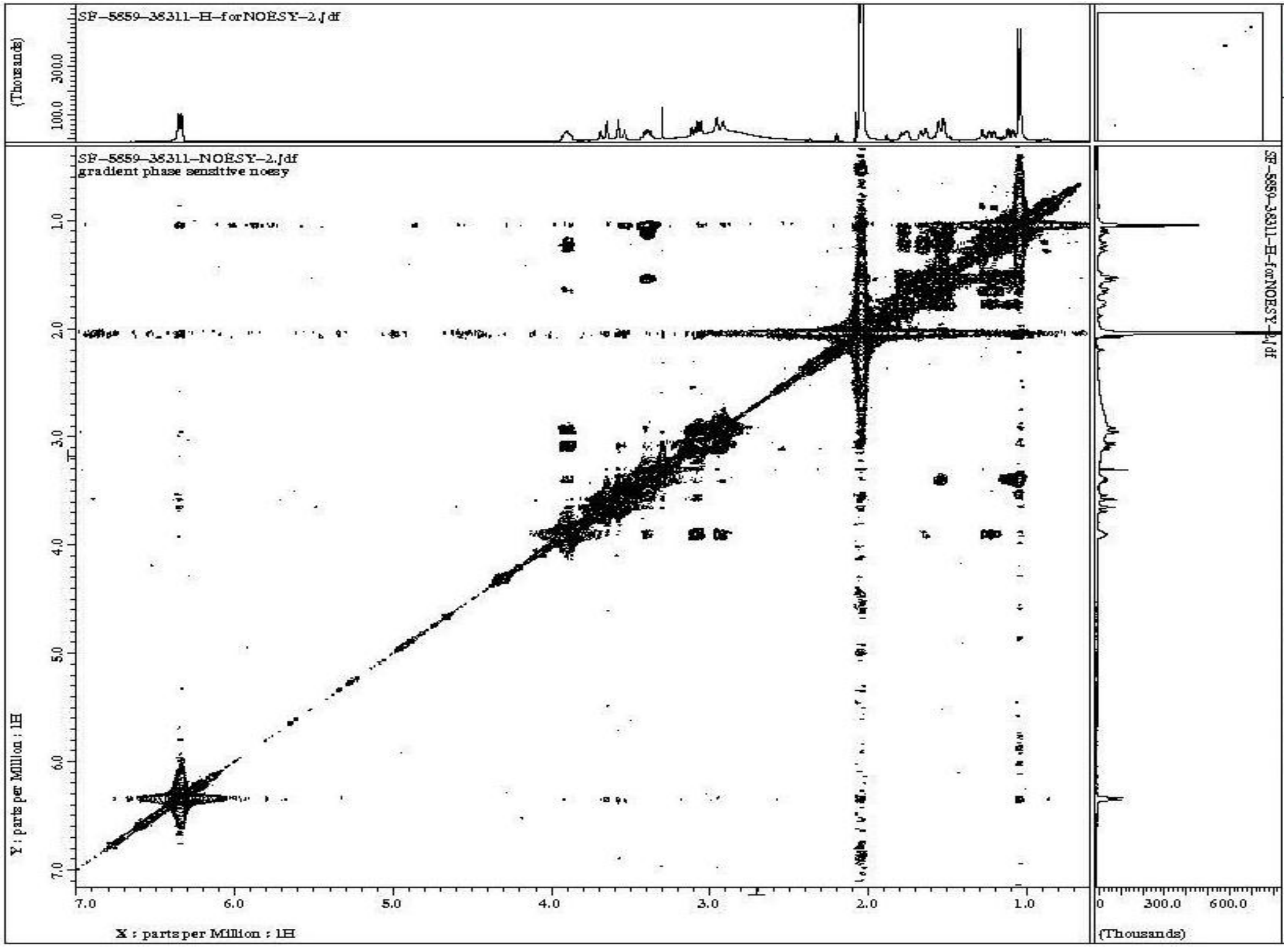

Figure S5. NOESY spectrum of compound **1**

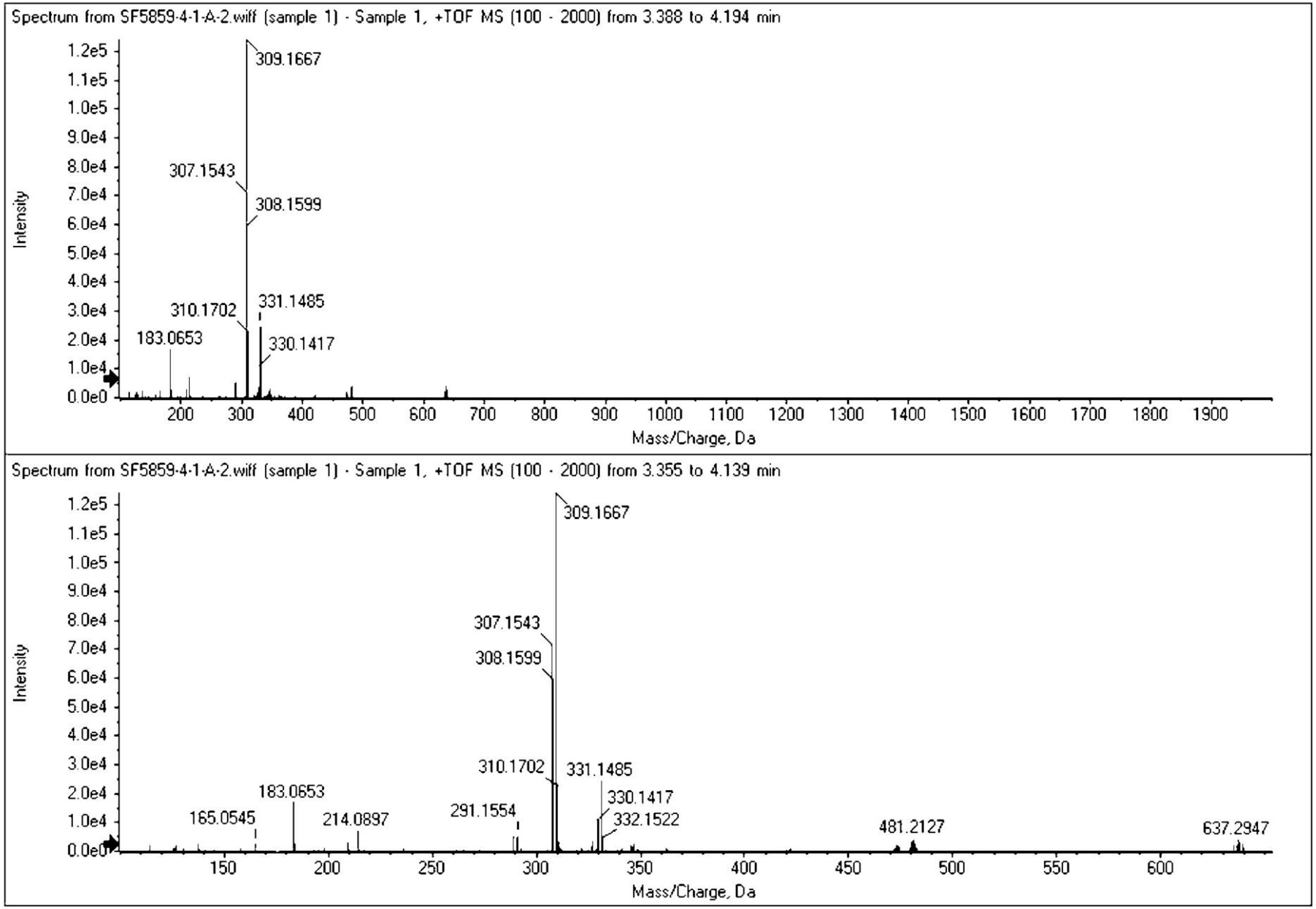

Figure S6. HRESI mass spectrum of compound **3a**

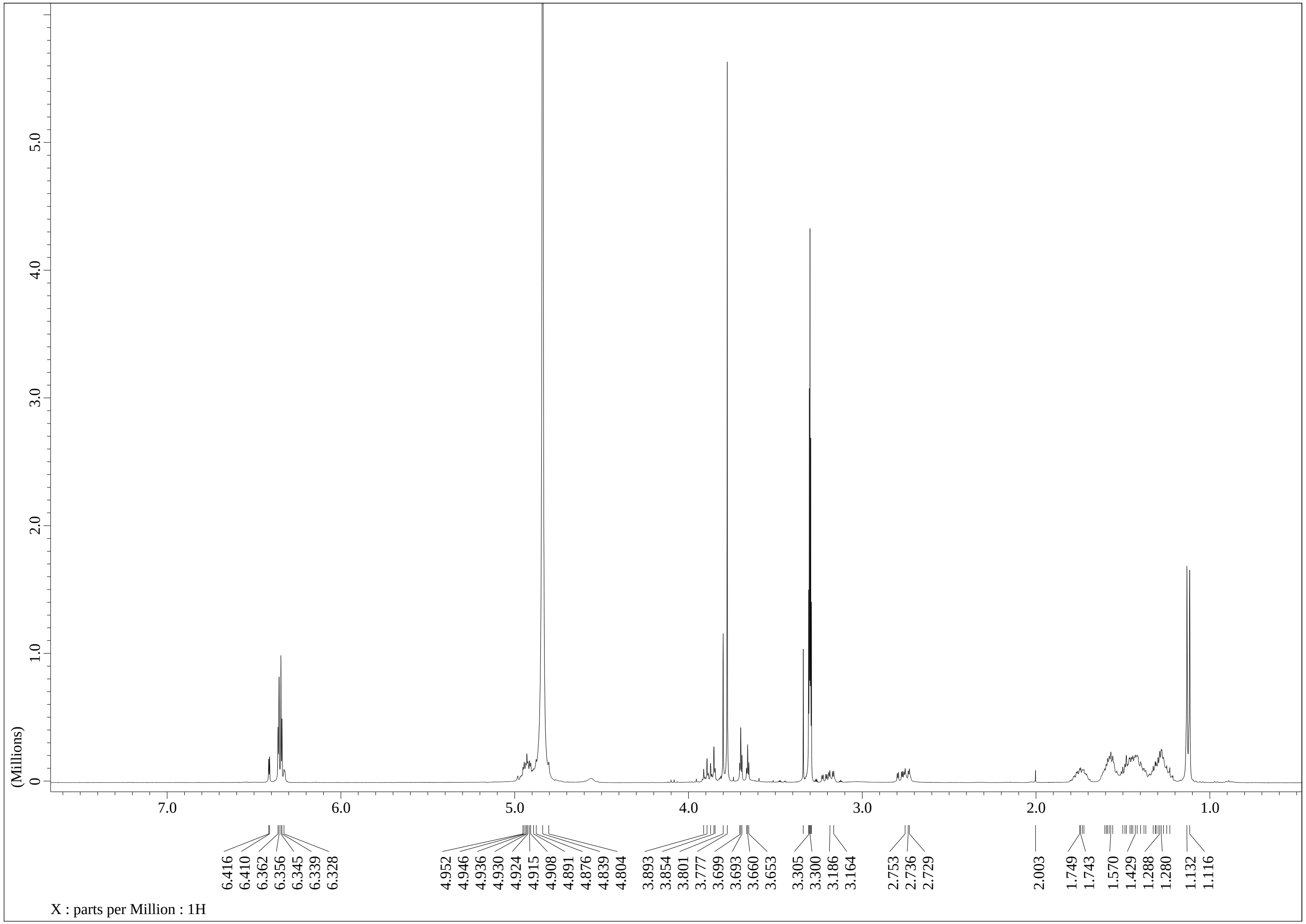

Figure S7. ^{1}H NMR spectrum (400 MHz, CD_3OD) of compound **3a**

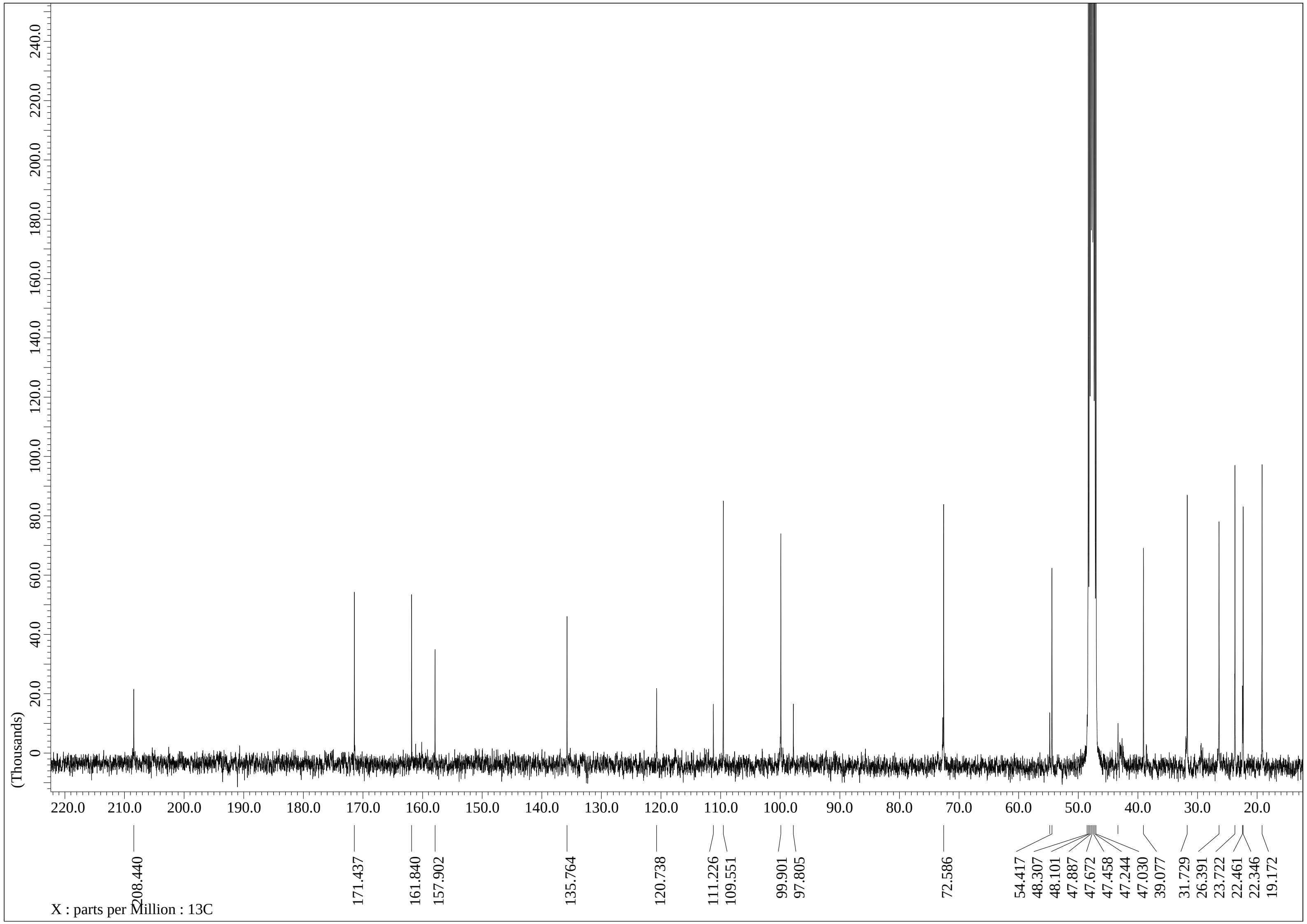

Figure S8. ^{13}C NMR spectrum (100MHz, CD_3OD) of compound **3a**

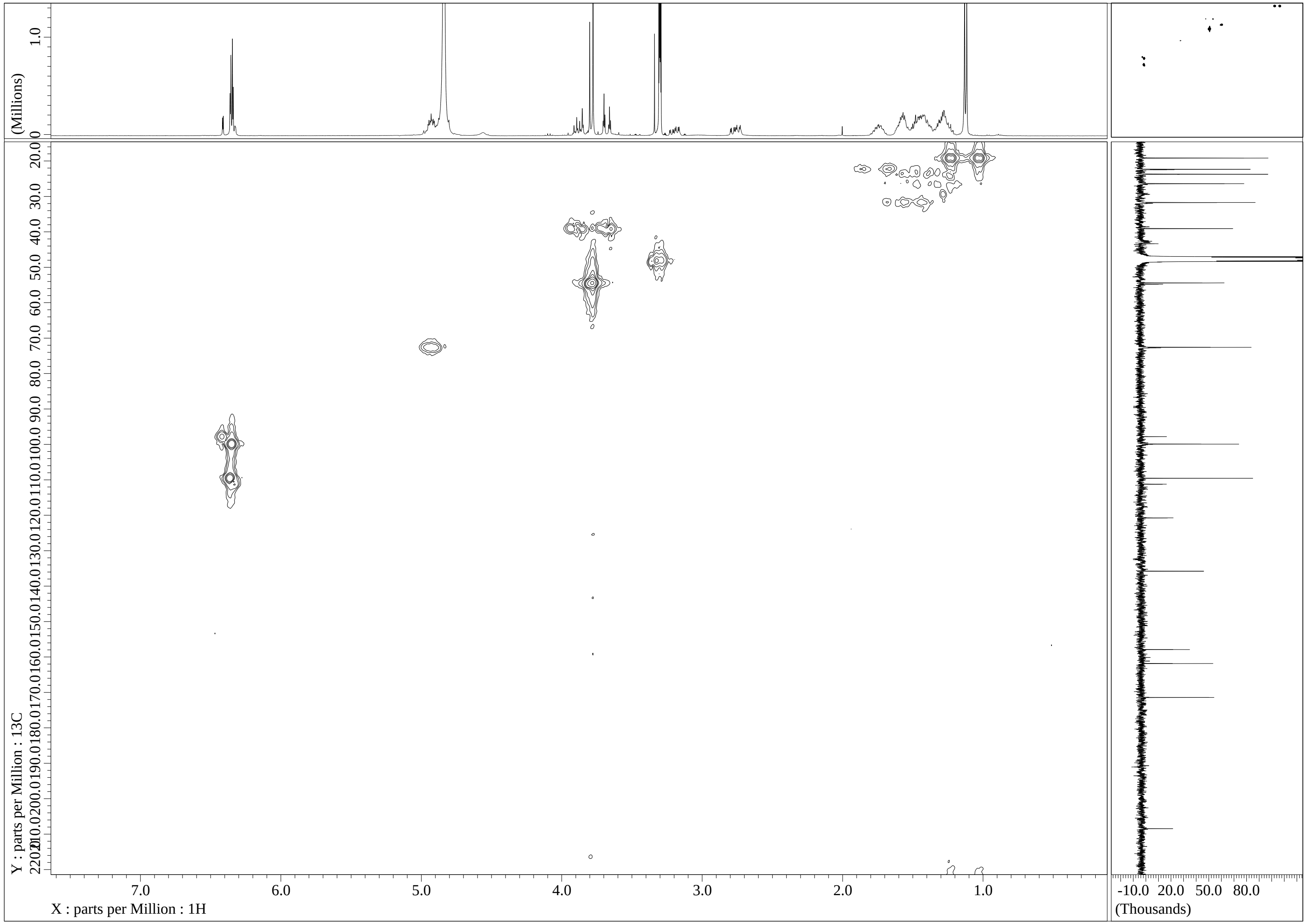

Figure S9. HMQC spectrum of compound **3a**

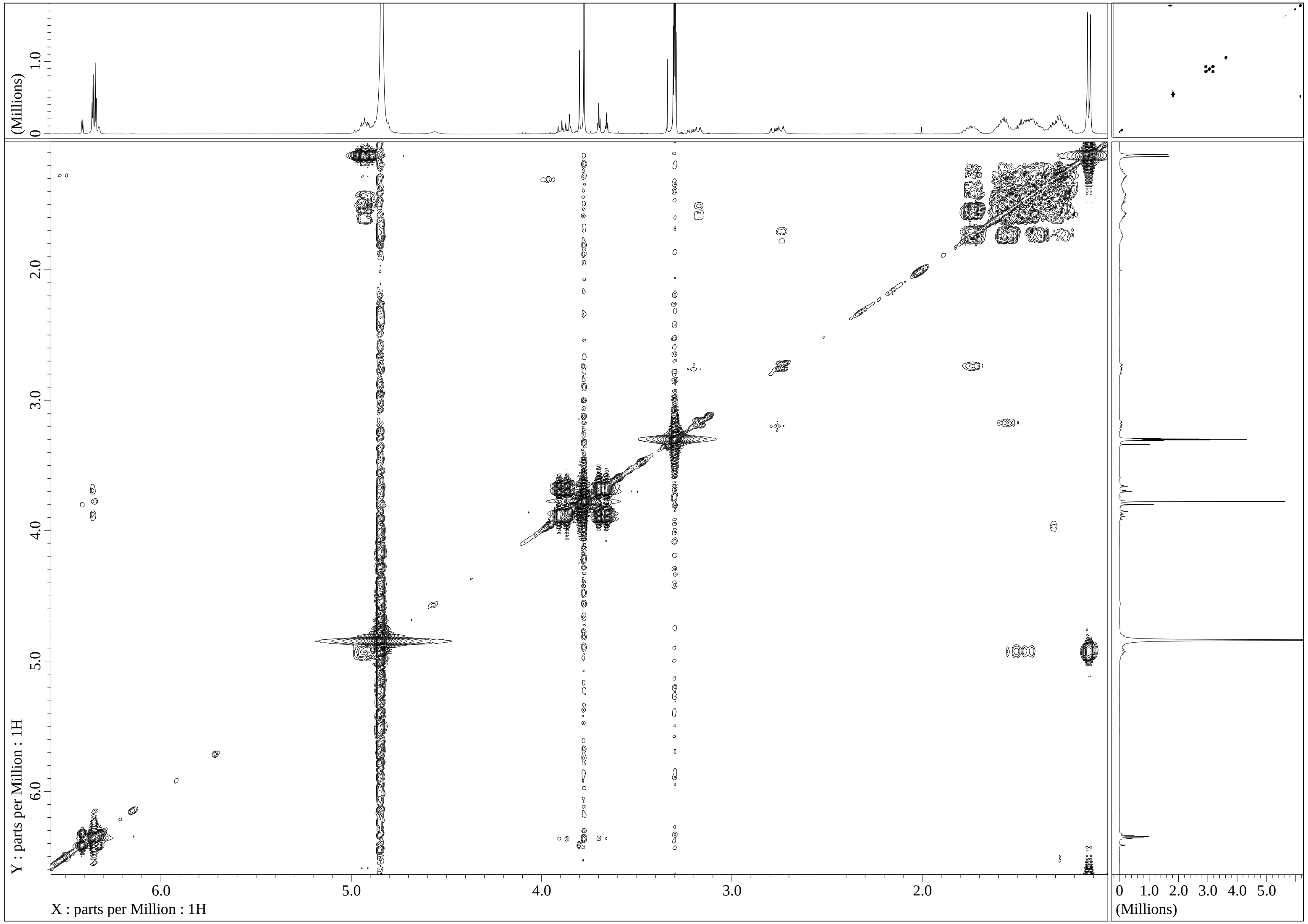

Figure S10. COSY spectrum of compound **3a**

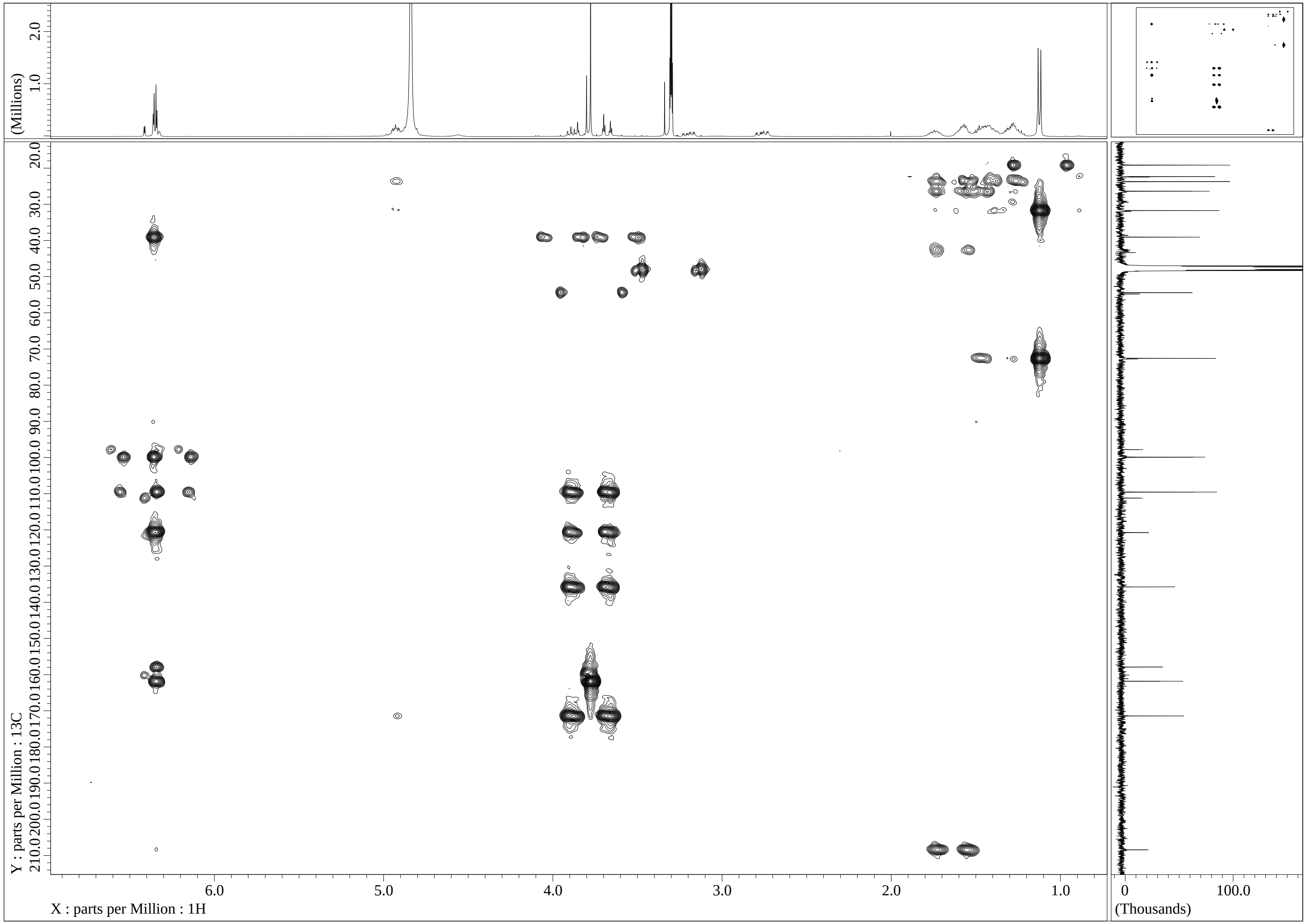

Figure S11. HMBC spectrum of compound **3a**

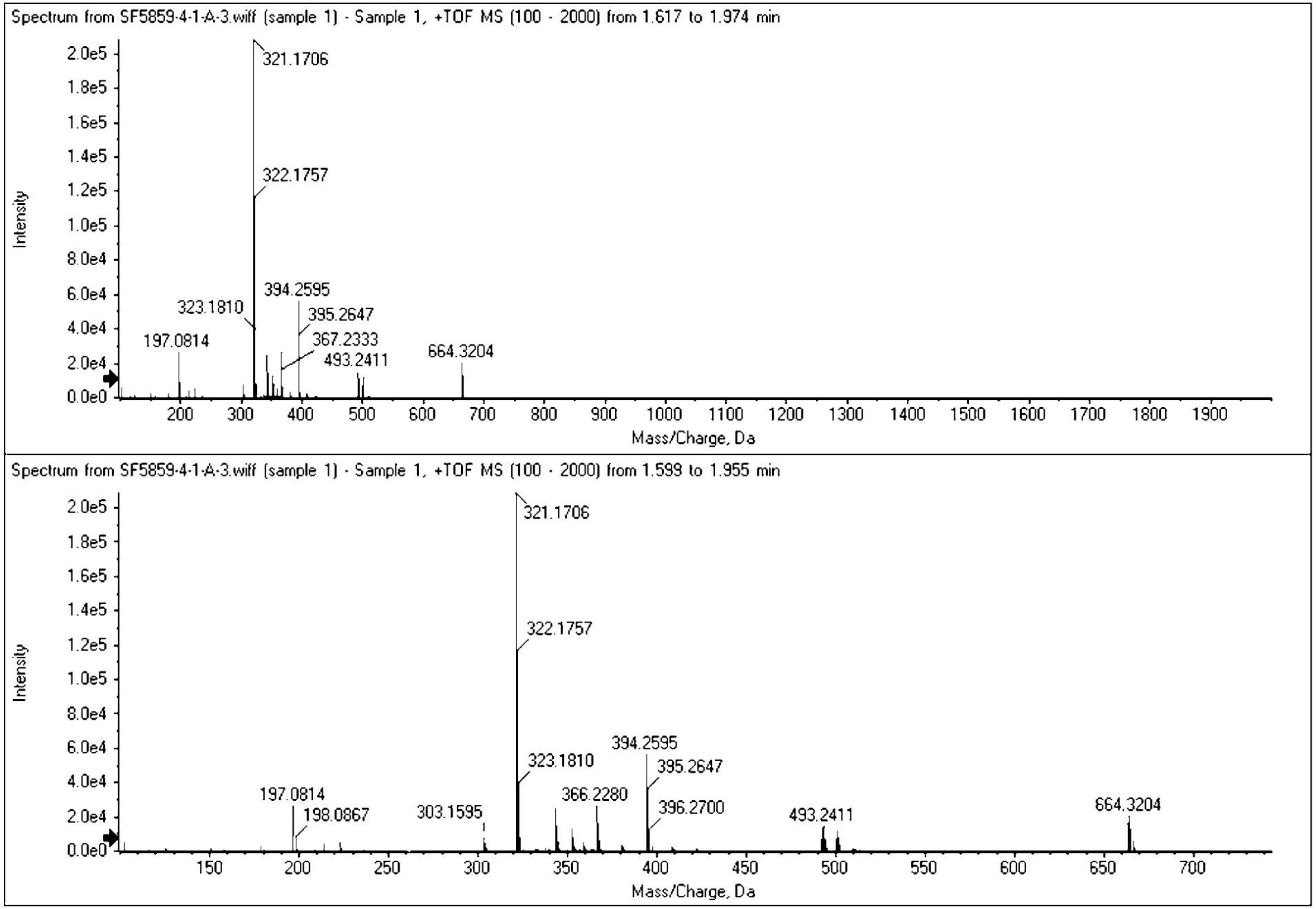

Figure S12. HRESI mass spectrum of compound **3b**

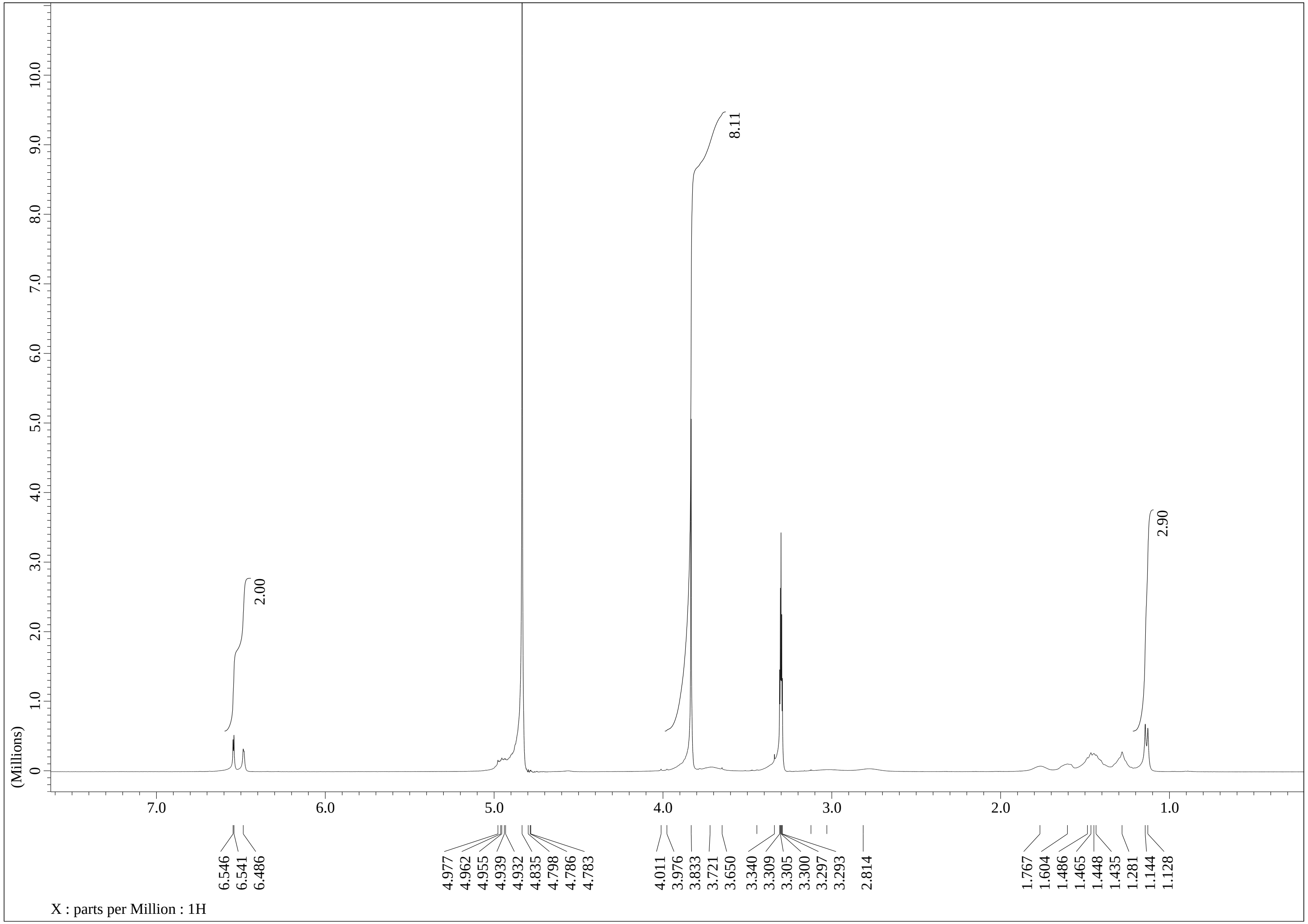

Figure S13. ^{1}H NMR spectrum (400 MHz, CD_3OD) of compound **3b**

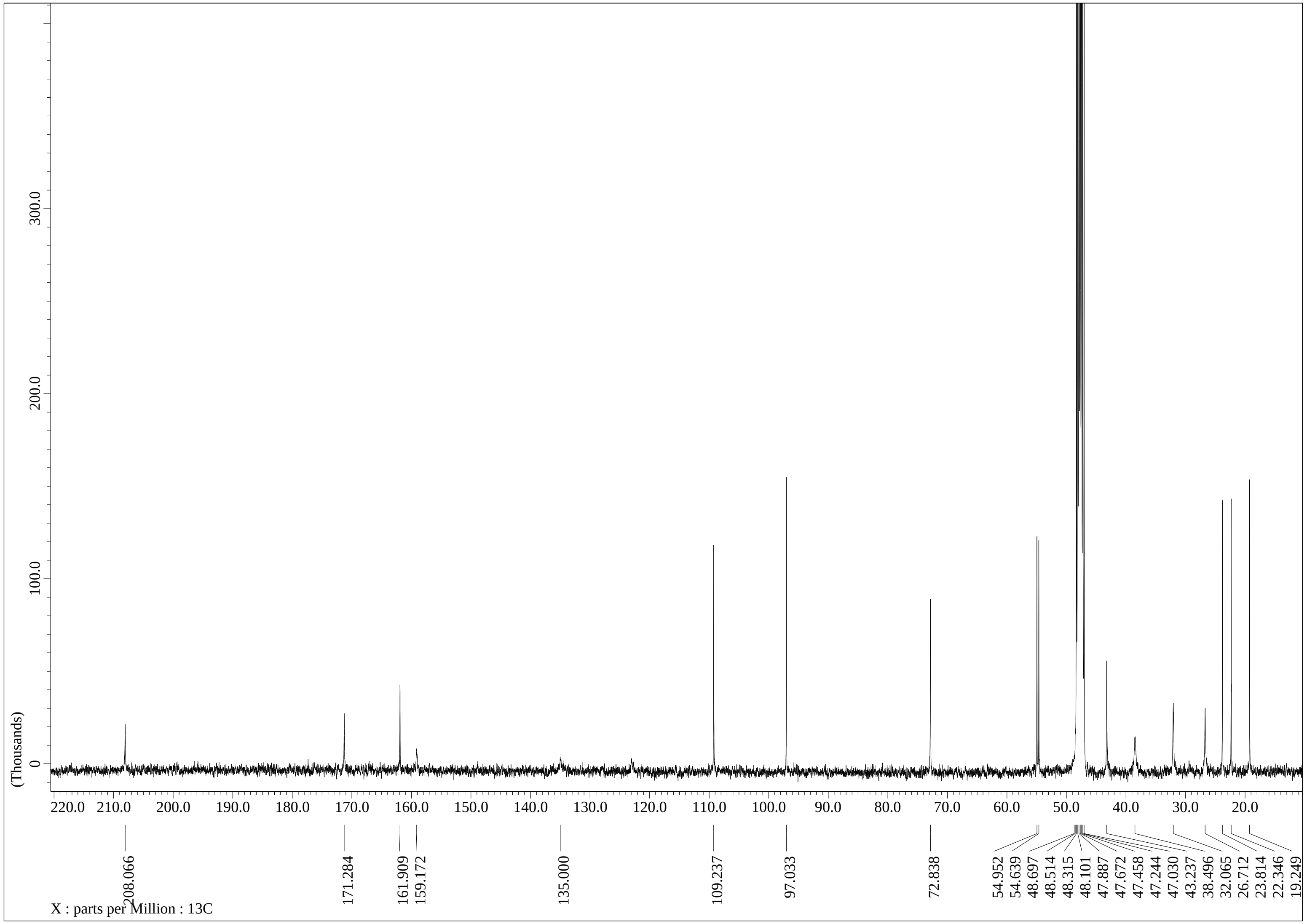

Figure S14. ^{13}C NMR spectrum (100 MHz, CD_3OD) of compound **3b**

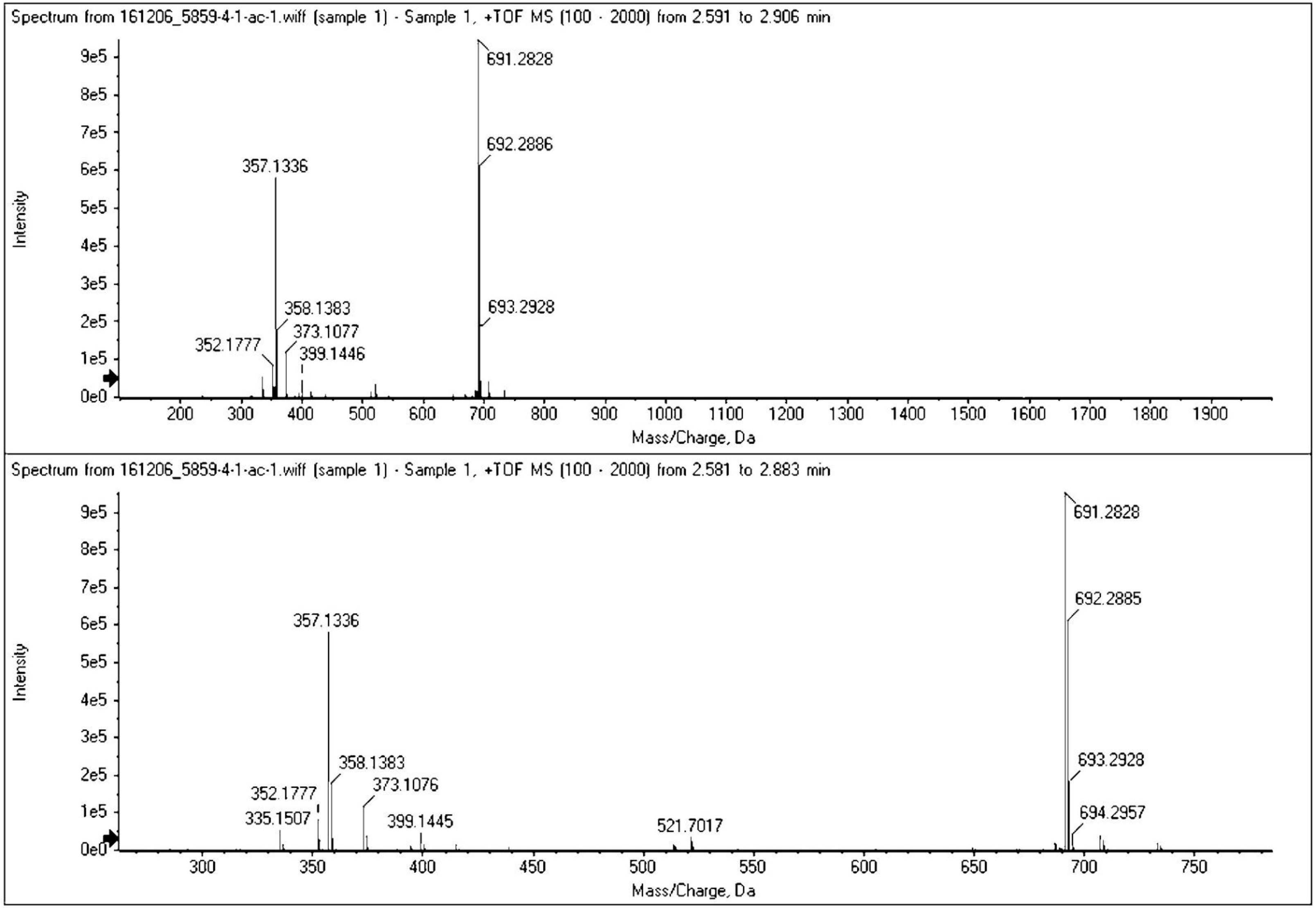

Figure S15. HRESI mass spectrum of compound **3c**

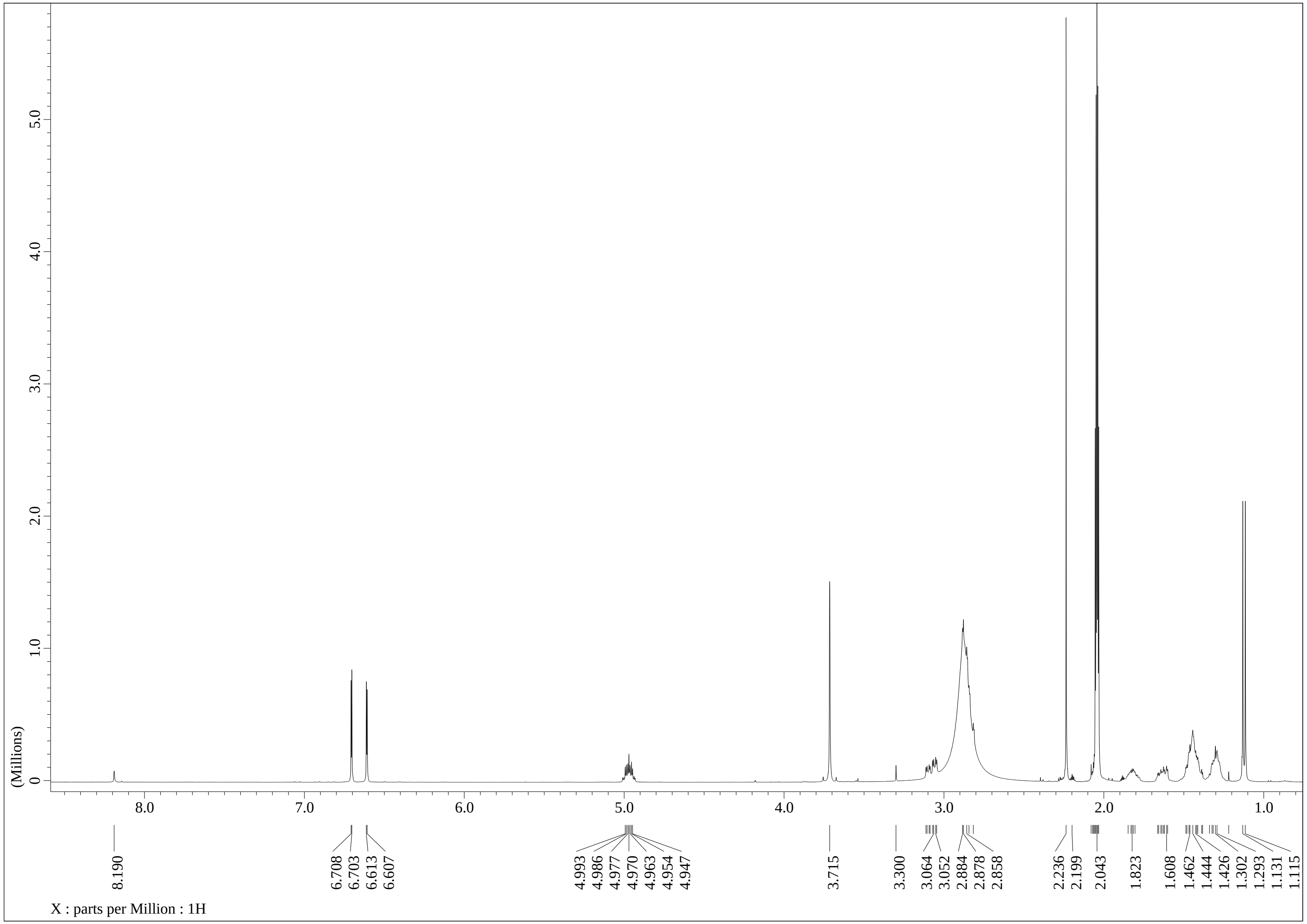

Figure S16. ^{1}H NMR spectrum (400 MHz, acetone-*d6*) of compound **3c**

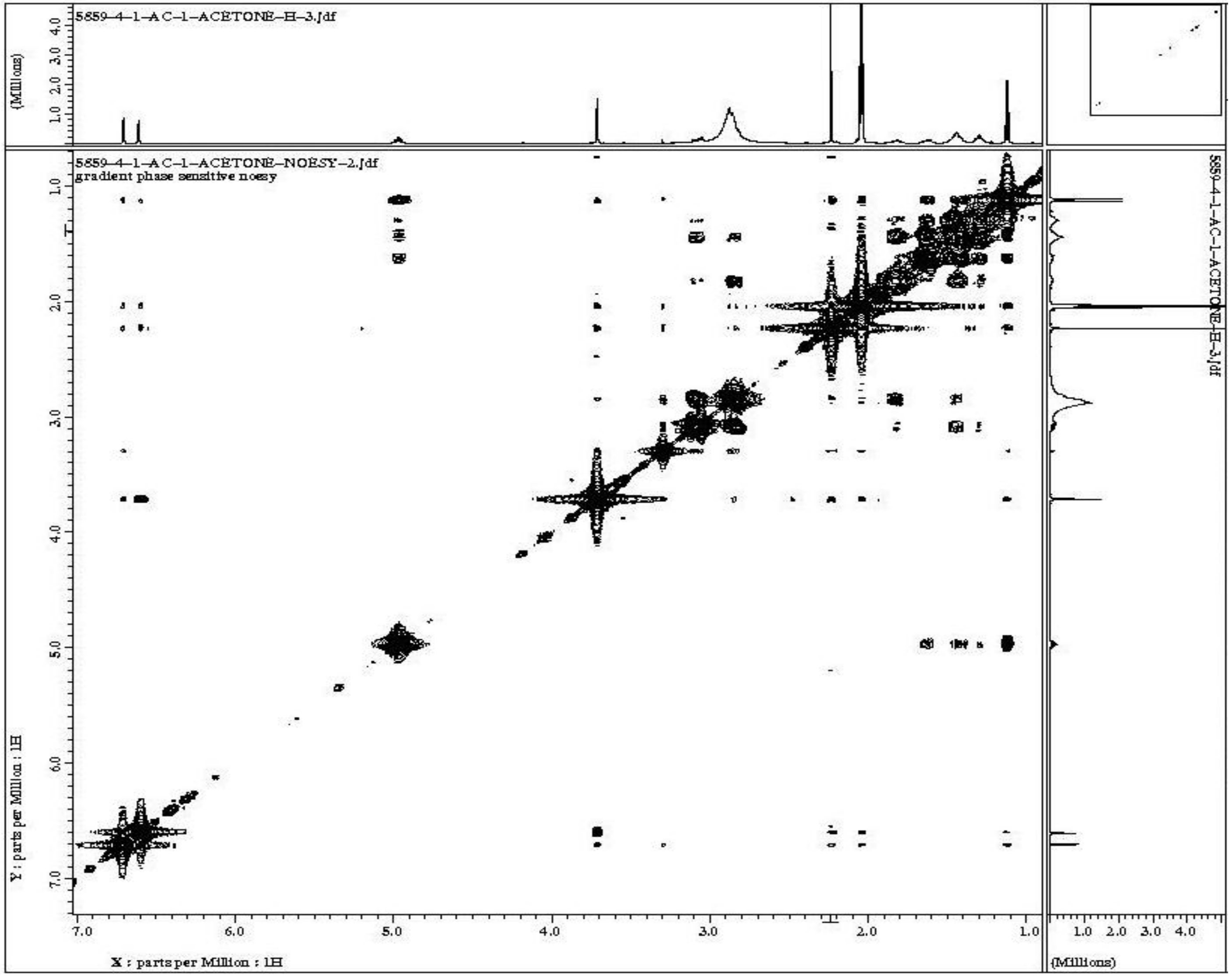

Figure S17. NOESY spectrum of compound **3c**

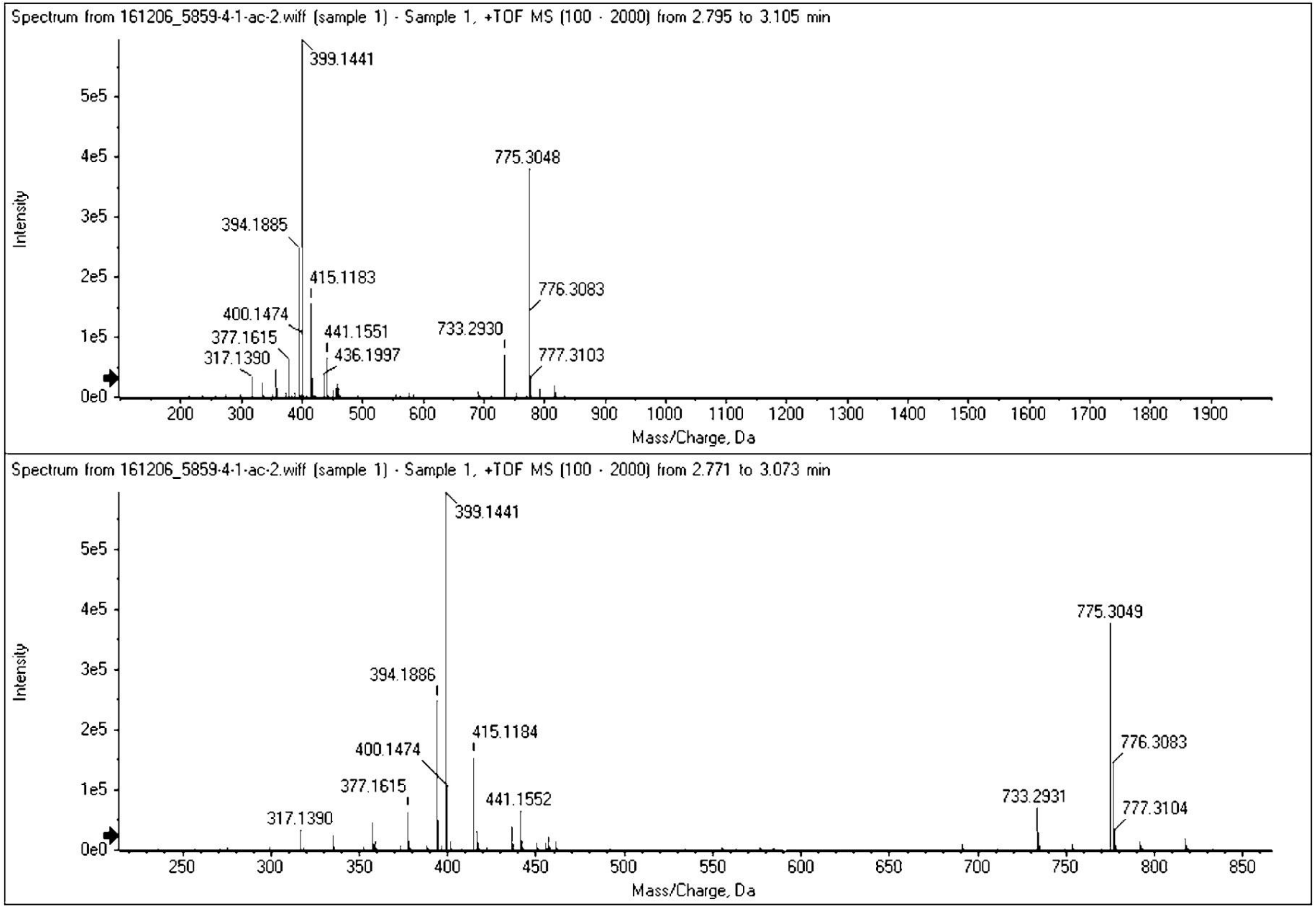

Figure S18. HRESI mass spectrum of compound **3d**

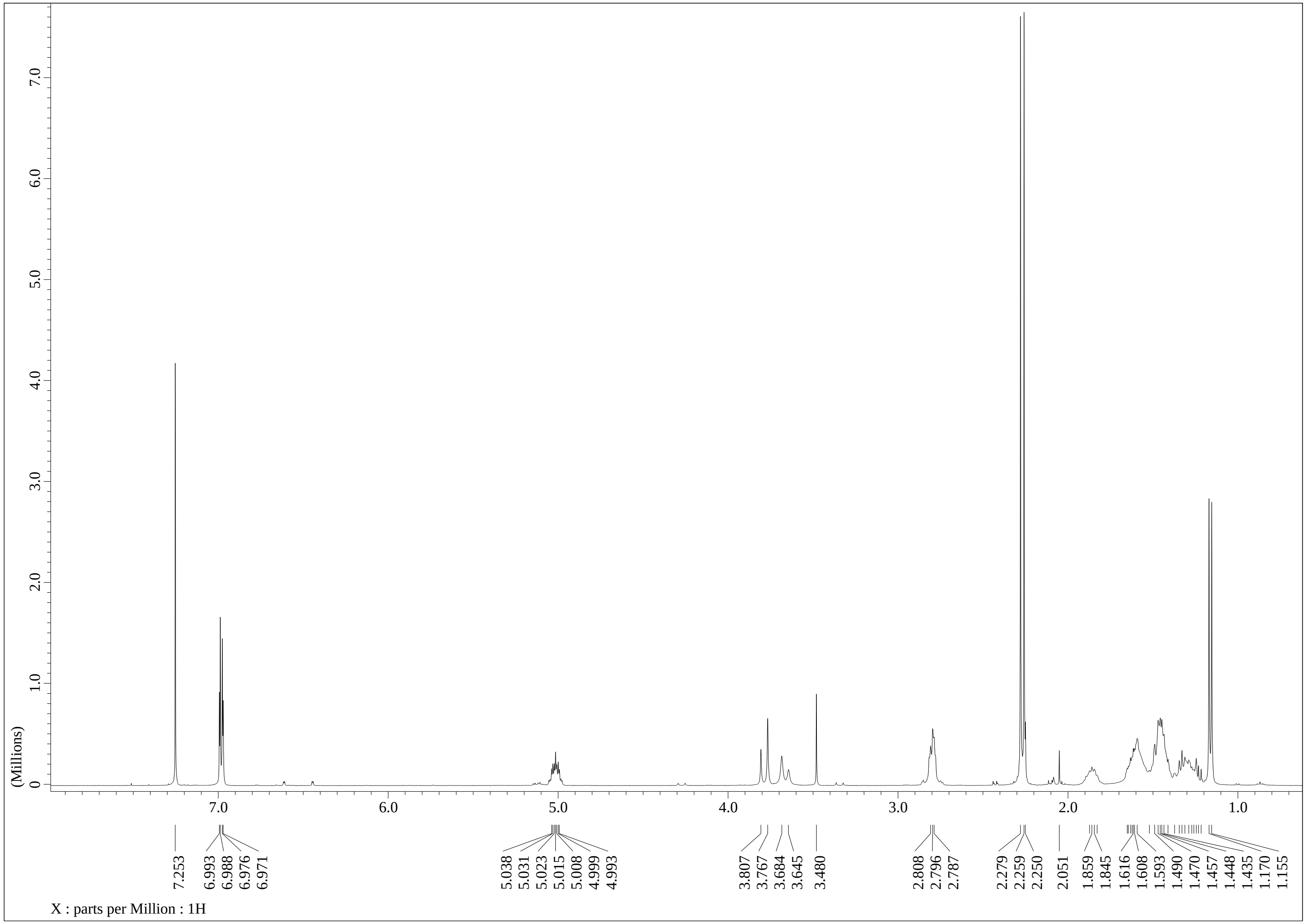

Figure S19. ^{1}H NMR spectrum (400 MHz, $CDCl_3$) of compound **3d**

Table S1. The optical rotation values of **1-9** in comparison with the published values.

Compounds	Optical rotation value, [α]D		
	measured value (EtOH)	literature value (EtOH)	literature value of enantiomer (EtOH)
Curvulone C (**1**)	+13.6 (c = 0.22)	-	-
Curvulone B (**2**)	-15.2 (c = 0.18)	-22.0 (c = 0.27)[1]	n.e
Curvularin (**3**)	-37.1 (c = 2.4)	-33.0 (c = 2.0)[2]	+32.6 (c = 0.75)[3]
(11*R*,15*S*)-11-hydrocurvularin (**4**)	-14.9 (c = 0.44)	- 29.4 (c = 0.33)[4]	+25.2 (c = 0.26)[4]
(11*S*,15*S*)-11-hydrocurvularin (**5**)	-54.6 (c = 0.56)	-10.9 (c = 0.19)[4]	+6.9 (c = 0.47)[4]
(11*R*,15*S*)-11-methoxycurvularin (**6**)	-7.1 (c = 0.43)	-4.0 (c = 0.3)[5]	n.e
(11*S*,15*S*)-11-methoxycurvularin (**7**)	-17.0 (c = 0.51)	-17 (c = 1.0)[5]	n.e
(10*E*,15*S*)-10,11-dehydrocurvularin (**8**)	-51.6 (c = 2.22)	-79.8 (c = 3.0)[4]	+79.1 (c = 0.4)[4]
(10*Z*,15*S**)-10,11-dehydrocurvularin (**9**)	-19.9 (c = 0.15)	+7.3 (c = 0.78)[6]	n.e

n.e: no enantiomer reported in the literature

References

1. Dai, J.; Krohn, K.; Flörke, U.; Pescitelli, G.; Kerti, G.; Papp, T.; Kövér, K.E.; Bényei, A. C.; Draeger, S.; Schulz, B.; Kurtán, T. Curvularin-type metabolites from the fungus *Curvularia* sp. isolated from a marine alga. *Eur. J. Org. Chem.* **2010**, *2010*, 6928–6937. [CrossRef]
2. Elzner, S.; Schmidt, D.; Schollmeyer, D.; Erkel, G.; Anke, T.; Kleinert, H.; Förstermann, U.; Kunz, H. Inhibitors of inducible NO synthase expression: total synthesis of (*S*)-curvularin and its ring homologues. *ChemMedChem* **2008**, *3*, 924–939. [CrossRef]
3. Bracher, F.; Schulte, B. An enantiodivergent synthesis of both enantiomers of the macrocyclic lactone curvularin. *Liebigs Annalen/Recueil* **1997**, *1997*, 1979–1982. [CrossRef]
4. Greve, H.; Schupp, P.J.; Eguereva, E.; Kehraus, S.; Kelter, G.; Maier, A.; Fiebig, H.-H.; König, G.M. Apralactone A and a new stereochemical class of curvularins from the marine fungus *Curvularia* sp. *Eur. J. Org. Chem.* **2008**, *2008*, 5085–5092. [CrossRef]
5. Liang, Q.; Sun, Y.; Yu, B.; She, X.; Pan, X. First total syntheses and spectral data corrections of 11-α-methoxycurvularin and 11-β-methoxycurvularin. *J. Org. Chem.* **2007**, *72*, 9846–9849. [CrossRef]

6. Lai, S.; Shizuri, Y.; Yamamura, S.; Kawai, K.; Terada, Y.; Furukawa, H. Novel curvularin-type metabolites of a hybrid strain ME 0005 derived from *penicillium* citreo-viride B. IFO 6200 and 4692. *Tetrahedron Lett.* **1989**, *30*, 2241–2244. [CrossRef]
